# Astrocytic Sirt2-Mediated Delactylation Limits Blood–Spinal Cord Barrier Reconstruction after Spinal Cord Injury

**DOI:** 10.34133/research.1447

**Published:** 2026-09-30

**Authors:** Yixue Hu, Hao Hu, Zhonghan Wu, Yiyang Mu, Zeqiang Chen, Linhan Yang, Mengdi Zhang, Zihao Chen, Zhenyu Zhang, Ziyu Li, Fei Yao, Shuisheng Yu, Dasheng Tian, Li Cheng

**Affiliations:** ^1^ Department of Orthopaedics, The Second Affiliated Hospital of Anhui Medical University, Hefei, China.; ^2^ Institute of Orthopaedics, Research Center for Translational Medicine, The Second Affiliated Hospital of Anhui Medical University, Hefei, China.

## Abstract

Restoration of the blood–spinal cord barrier (BSCB) is required for substantial neurological improvement after spinal cord injury (SCI). Astrocytes are integral to this barrier and, after injury, undergo glycolytic adaptation accompanied by lactate accumulation. Whether a lactate-linked modification program in astrocytes participates in BSCB repair has remained unresolved. Here, we identify astrocytic delactylation as a brake on BSCB repair. Sirtuin2 (Sirt2), a delactylase, was strongly induced in astrocytes at the lesion border, and its expression paralleled the pathological expansion of microvessels. Selective deletion of *Sirt2* in astrocytes increased lactylation, produced a lactate-rich metabolic state, attenuated inflammatory astrocyte programs, enhanced repair-associated features, and reinforced BSCB repair. These changes further reduced inflammatory infiltration and promoted neuronal retention, increased 5-hydroxytryptamine-positive fiber density, and improved locomotor recovery. Pharmacological Sirt2 inhibition produced similar vascular and locomotor benefits. Mechanistically, *Sirt2* loss elevated H4K8la occupancy at the hepatocyte growth factor (*Hgf*) locus and promoted *Hgf* transcription, thereby facilitating BSCB repair. Astrocyte-specific *Sirt2* ablation also weakened CD8^+^ T-cell-related inflammatory activity after SCI. Collectively, our findings position astrocytic *Sirt2*-dependent delactylation as a negative epigenetic–metabolic regulator of BSCB reconstruction and support astrocytic delactylation as a therapeutic target for SCI.

## Introduction

Spinal cord injury (SCI) often causes persistent deficits in motor, sensory, and autonomic function and places major clinical and socioeconomic burdens on patients and society [1,2]. The immediate mechanical impact is followed by secondary injury cascades, including blood–spinal cord barrier (BSCB) disruption, neurovascular dysfunction, inflammation, and neural tissue damage [2–8]. Although some endogenous repair is initiated after the acute phase, the barrier architecture does not fully return to its pre-injury state [5]. Basement membrane injury and incomplete restoration of endothelial junctional proteins contribute to persistent pathological vascular remodeling [5,9]. Consequently, vessels in chronic lesions frequently remain dilated and permeable, which sustains barrier failure [9–11]. Leakage through these abnormal vessels sustains inflammation and secondary tissue damage, thereby impeding neural repair [12,13]. Thus, effective SCI therapy should address not only inflammation, scarring, and trophic support but also reconstitution of vascular integrity and BSCB function [10,14]. However, the mechanisms that govern effective BSCB reconstruction after SCI remain insufficiently understood [5,15].

The BSCB contains nonfenestrated endothelial cells, pericytes embedded along the basement membrane, and astrocytic end feet that wrap the vascular wall [14]. Astrocytes support neural homeostasis and help maintain endothelial barrier properties under physiological conditions [16,17]. After injury, however, their influence on the vasculature depends strongly on cellular state and local context [18–22]. Some reactive astrocyte programs damage endothelial junctions by releasing cytokines such as interleukin-6 and by promoting leukocyte recruitment [19,23], whereas other astrocyte states facilitate barrier repair after neural damage [21,22]. These observations highlight astrocytes as pivotal regulators of post-SCI BSCB repair, but the molecular switches that determine protective versus detrimental astrocyte behavior remain poorly defined.

In an injured central nervous system (CNS), astrocytes undergo marked metabolic reprogramming while participating in homeostasis, synaptic regulation, tissue repair, and control of axonal excitability [24,25]. This issue is especially relevant to SCI, where glucose uptake and utilization, together with broader energy metabolism, are disrupted [26,27]. Compared with many neural cell types, injured astrocytes show a pronounced shift toward glycolysis and generate more lactate [28]. Lactate is not merely an end product of glycolysis; it also provides a substrate for histone lactylation, a posttranslational modification that connects metabolic status to transcriptional output [29–31]. Emerging evidence indicates that astrocytic lactylation can exert context-dependent effects in different forms of CNS injury [32,33]. Lactylation levels are dynamically balanced by lactyltransferases and delactylases [34–39]. Most studies in CNS disorders have emphasized lactyltransferase-mediated histone or nonhistone lactylation, leaving astrocytic delactylation largely unexplored [40–44].

In this work, we show that astrocytic delactylation is a key determinant of BSCB repair after SCI through an epigenetic mechanism not previously established in this context. We found that sirtuin2 (Sirt2) is induced in reactive astrocytes after injury and is linked to pathological vascular remodeling. Astrocyte-specific deletion of *Sirt2* enhanced repair-associated astrocyte programs, promoted BSCB repair, and improved locomotor recovery. Mechanistically, Sirt2 regulated astrocytic hepatocyte growth factor (*Hgf*) transcription through H4K8la-dependent epigenetic control. *Sirt2* conditional knockout (cKO) also attenuated inflammatory responses associated with CD8^+^ T cells following SCI. Together, these data reveal an underappreciated role of astrocytic delactylation in neurovascular repair and identify Sirt2 as a candidate target for improving vascular and neural recovery.

## Results

### Astrocytic delactylation is associated with abnormal microvascular enlargement after SCI

We began by mapping how vascular pathology evolves across space and time after SCI using a mouse T10 spinal cord crush injury model. Previous studies have shown that vascular enlargement occurs in the astrocyte-poor lesion core (LC) [9,45,46]. Costaining for CD31 and glial fibrillary acidic protein (GFAP) showed that vessel enlargement was not confined to the previously described astrocyte-poor LC. Marked microvascular enlargement was also observed within the reactive astrocyte-rich marginal region at 3, 7, 14, and 28 d post-injury (dpi). By contrast, distant intact tissue, which contained few reactive astrocytes, showed little evidence of vascular enlargement (Fig. 1A to C). Measurement of vessel diameter revealed a progressive increase in the marginal region, peaking at 14 dpi and remaining elevated at 28 dpi (Fig. 1D). Because sustained dilation is a feature of pathological vascular remodeling, these findings indicate that SCI induces persistent vascular abnormalities accompanied by chronic BSCB dysfunction [9,10]. The concentration of this pathology in the reactive astrocyte-rich marginal region suggested that reactive astrocytes could contribute to pathological vascular remodeling after injury.

**Fig. 1.**
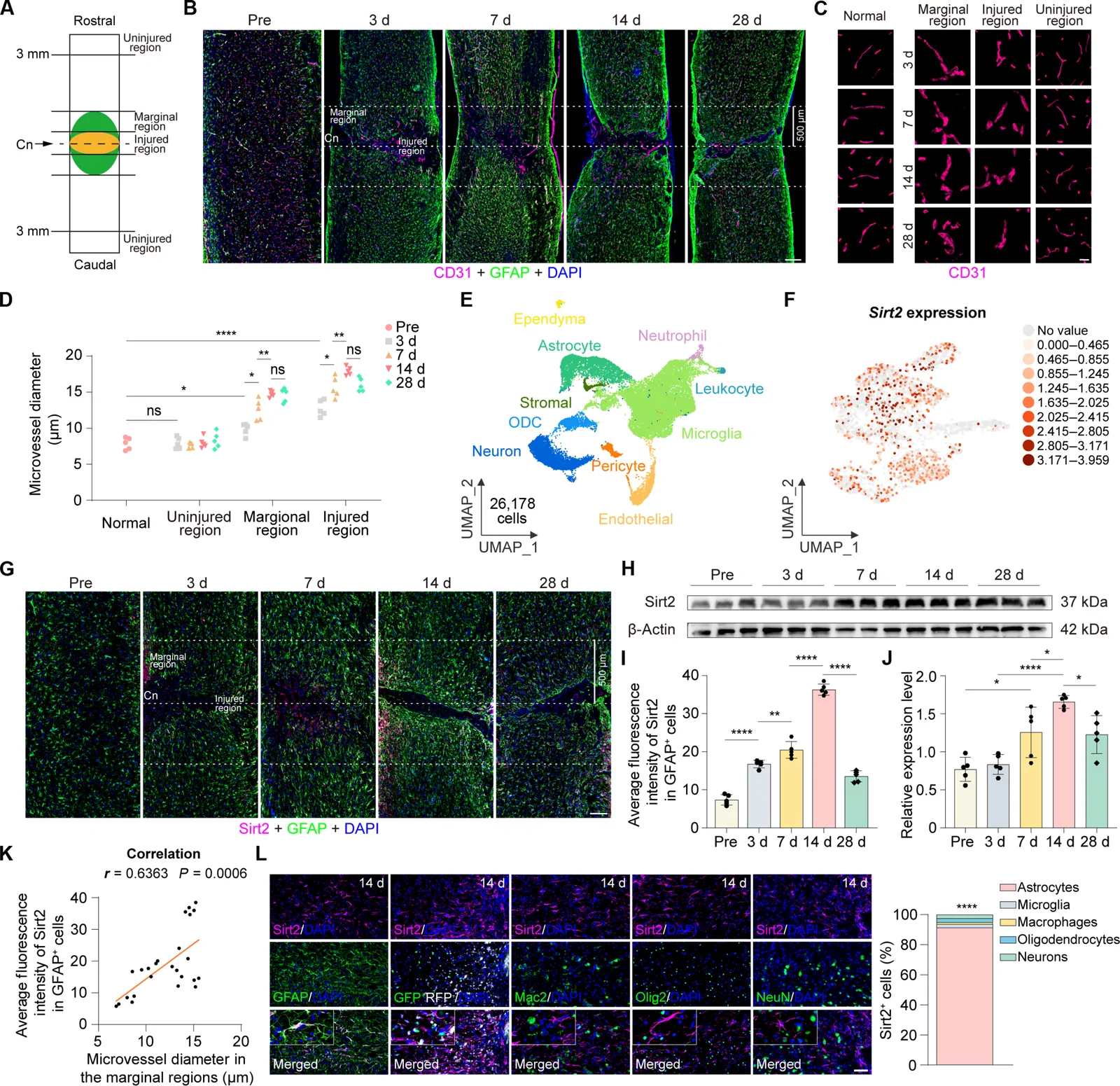
Astrocytic sirtuin2 (Sirt2) increases after spinal cord injury (SCI) and is associated with microvascular dilation. (A) Diagram of the lesion core (LC), marginal region, and uninjured area used for vascular analysis. (B to D) CD31 staining and microvessel diameter in the indicated regions before injury (Pre) and at 3, 7, 14, and 28 d post-injury (dpi). Scale bars, 200 μm in (B) and (20) μm in (C). (E and F) Uniform manifold approximation and projection (UMAP) of major cell populations and Sirt2 expression in reclustered astrocytes from a published SCI single-cell RNA sequencing (scRNA-seq) dataset. (G) Sirt2 (purple), glial fibrillary acidic protein (GFAP; green), and 4′,6-diamidino-2-phenylindole (DAPI; blue) staining at different time points. Scale bar, 200 μm. (H) Representative western blots of Sirt2 and β-actin in magnetically isolated spinal cord astrocytes collected at the indicated time points. (I) Corresponding quantification of Sirt2 fluorescence intensity in GFAP-positive astrocytes shown in (G). (J) Densitometric quantification of Sirt2 protein normalized to the corresponding β-actin signal. (K) Pearson correlation analysis between Sirt2 fluorescence intensity in GFAP^+^ astrocytes and microvessel diameter in the marginal region. (L) Sirt2 localization at 14 dpi by costaining with GFAP (astrocytes), GFP^+^/RFP^−^ (microglia), Mac2 (macrophages), Olig2 (oligodendrocytes), or NeuN (neurons). DAPI labels nuclei. Scale bars, 50 μm for overview images and 10 μm for insets. Data are mean ± standard error of the mean (SEM); *n* = 5 for (D), (I), (J), and (L). For the western blot analysis in (J), 5 biological replicates were analyzed at each time point, with each replicate comprising astrocytes pooled from 5 mice. One-way analysis of variance (ANOVA) with Tukey’s test was used for (D), (I), (J), and (L); Pearson correlation was used for (K). ns, not significant; *P < 0.05; **P < 0.01; ****P < 0.0001. GFP, green fluorescent protein; RFP, red fluorescent protein.

Because reactive astrocytes are major BSCB constituents and produce lactate after SCI, we next asked whether delactylation in astrocytes might relate to the vascular changes observed in the marginal zone [14,28,33]. We reanalyzed a published single-cell RNA sequencing (scRNA-seq) dataset from injured spinal cord (Fig. 1E and Fig. S1A) [47]. After isolating and reclustering astrocytes, we examined sirtuin genes together with classical histone deacetylases reported to exhibit delactylase activity [38,39,48]. Among all candidates, Sirt2 was the most enriched delactylase in spinal cord astrocytes after injury (Fig. 1F and Fig. S1B and C).

We then determined the temporal pattern of Sirt2 in astrocytes after SCI. Immunofluorescence revealed a rapid rise in Sirt2 signal in the GFAP^+^ astrocytes of the marginal region, with the highest level at 14 dpi (Fig. 1G and I). Densitometric analysis of Sirt2 immunoblots from magnetically isolated astrocytes confirmed the same temporal pattern, with Sirt2 protein levels peaking at 14 dpi and subsequently declining (Fig. 1H and J). Reanalysis of the single-cell dataset showed a similar temporal profile (Fig. S1D). Importantly, the temporal pattern of Sirt2 induction was associated with the progression of microvascular dilation (Fig. 1D, I, and J and Fig. S1D). Pearson correlation analysis further showed that stronger astrocytic Sirt2 signal was associated with a larger microvessel diameter in the marginal region (*r* = 0.6363, *P* = 0.0006; Fig. 1K). Cell-type localization showed that Sirt2-positive cells were predominantly GFAP^+^ astrocytes and were rare among microglia, macrophages, oligodendrocytes, and neurons (Fig. 1L). Thus, Sirt2 is an astrocyte-enriched delactylase whose post-injury induction is closely associated with pathological vascular remodeling after SCI.

### Astrocytic Sirt2 deletion enhances lactylation and promotes metabolic reprogramming after SCI

To assess the functional relevance of astrocytic delactylation after SCI, we produced tamoxifen-inducible *Aldh1l1^CreERT2^*-mediated *Sirt2* cKO mice by crossing *Sirt2^flox/flox^* animals with *Aldh1l1^CreERT2^* mice, hereafter referred to as *Sirt2* cKO mice (Fig. 2A) [49,50]. Mutant mice grew normally and showed no baseline behavioral abnormalities (Fig. S2A to D). After tamoxifen induction, immunofluorescence showed a marked reduction in Sirt2 protein levels within GFAP-positive cells at 14 dpi (Fig. 2B and C).

**Fig. 2.**
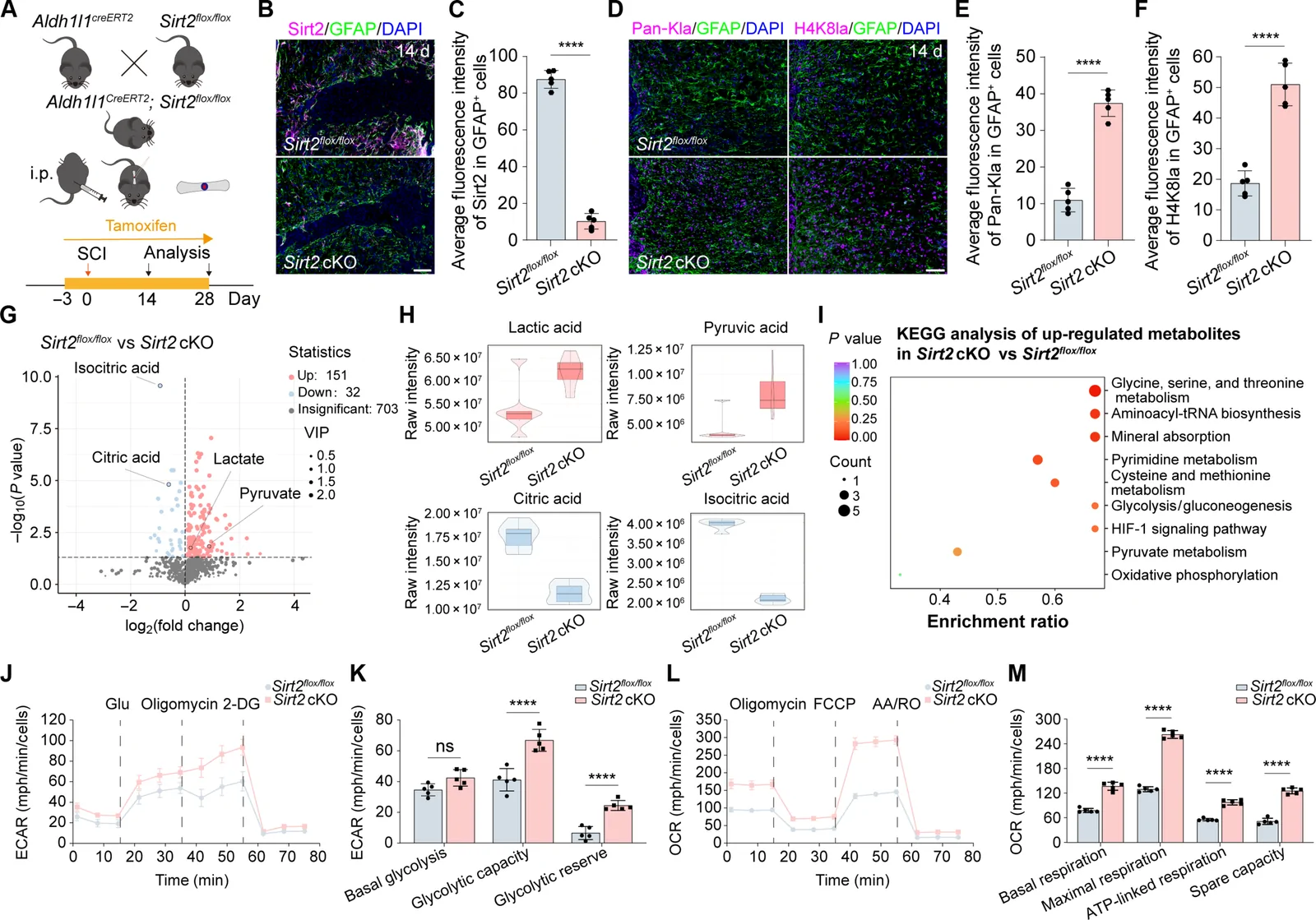
Astrocytic sirtuin2 (Sirt2) deletion increases lactylation and changes metabolic activity after spinal cord injury (SCI). (A) Breeding and tamoxifen-induction scheme for *Aldh1l1^CreERT2^*-mediated *Sirt2* conditional knockout (cKO) mice. (B and C) Sirt2 (purple), glial fibrillary acidic protein (GFAP; green), and 4′,6-diamidino-2-phenylindole (DAPI; blue) staining and quantification in injured spinal cord at 14 d post-injury (dpi). Scale bar, 50 μm. (D to F) Representative GFAP (green), Pan-Kla or H4K8la (purple), and DAPI (blue) staining, with quantification in GFAP^+^ astrocytes at 14 dpi. Scale bar, 50 μm. (G and H) Untargeted metabolomics of purified astrocytes showing selected altered metabolites. (I) Kyoto Encyclopedia of Genes and Genomes (KEGG) enrichment of metabolites increased in *Sirt2* cKO astrocytes. (J and K) Seahorse extracellular acidification rate (ECAR) profile and related parameters. (L and M) Seahorse oxygen consumption rate (OCR) profile and related parameters. Data are mean ± standard error of the mean (SEM); *n* = 5 for (C), (E), (F), (K), and (M); *n* = 6 for (G) and (H). A 2-tailed unpaired Student *t* test was used. ns, not significant; ****P < 0.0001.

Given the delactylase activity of Sirt2, we examined whether its loss in astrocytes altered lactylation after SCI [39]. Pan-lysine lactylation staining revealed a marked increase in global lactylation in GFAP^+^ astrocytes from *Sirt2* cKO mice compared with controls (Fig. 2D and E). We also measured H4K8la, a lactylation mark implicated in astrocytic transcriptional regulation and reported to be controlled by *Sirt2* [39,51]. H4K8la was similarly increased in *Sirt2*-deficient astrocytes (Fig. 2D and F). These data indicate that astrocytic *Sirt2* restrains both total lysine lactylation and H4K8la accumulation after SCI.

We next investigated the metabolic consequences of *Sirt2* deletion in astrocytes by conducting untargeted metabolomics on purified spinal cord astrocytes at 14 dpi. Compared with controls, *Sirt2* cKO astrocytes contained higher amounts of lactate and pyruvate but lower levels of citrate and isocitrate, 2 tricarboxylic acid cycle intermediates (Fig. 2G and H and Fig. S2E). Kyoto Encyclopedia of Genes and Genomes (KEGG) enrichment analysis of up-regulated metabolites in Sirt2 cKO astrocytes highlighted glycolysis/gluconeogenesis, pyruvate metabolism, HIF-1 signaling, and oxidative phosphorylation (Fig. 2I). Together with the rise in lactate and pyruvate, these changes indicate enhanced glycolytic output and a lactate-enriched metabolic state favorable for lactylation.

Seahorse extracellular flux analysis showed that *Sirt2* cKO astrocytes displayed higher extracellular acidification rate and oxygen consumption rate profiles than control astrocytes (Fig. 2J and L). Quantification demonstrated increases in glycolytic capacity, glycolytic reserve, basal respiration, maximal respiration, adenosine triphosphate (ATP)-linked respiration, and spare respiratory capacity (Fig. 2K and M), whereas basal glycolysis showed an upward trend that did not reach statistical significance (Fig. 2K). Thus, astrocytic *Sirt2* deletion enhances both glycolytic function and overall bioenergetic capacity.

Overall, the loss of *Sirt2* in astrocytes creates 2 cooperating conditions that promote lactylation: increased lactate availability and removal of delactylase activity. This combination favors the buildup of lactylation marks, including H4K8la, after SCI.

### Sirt2 deletion in astrocytes attenuates inflammatory programs and enhances repair-associated features in reactive astrocytes

To determine how astrocytic *Sirt2* deletion reshapes the lesion milieu, we performed scRNA-seq on spinal cord tissue harvested at 14 dpi (Fig. 3A) [52,53]. After tissue dissociation, barcoding, and sequencing, 34,827 cells were recovered from control and *Sirt2* cKO samples. Unsupervised clustering resolved 10 major populations: microglia, monocyte-derived macrophages, oligodendrocyte-lineage cells, monocytes, lymphocytes, endothelial cells, astrocytes, fibroblasts, pericytes, and neutrophils (Fig. 3B and Fig. S3A). Cell identities were assigned according to canonical markers and verified against independent reference datasets (Fig. S4A to K) [54–56]. Cell-composition analysis showed fewer lymphocytes and more endothelial cells in *Sirt2* cKO spinal cords, whereas the overall astrocyte fraction changed only modestly (Fig. S5A to C). These findings indicate that astrocytic *Sirt2* deficiency modifies the broader lesion environment without substantially altering astrocyte abundance.

**Fig. 3.**
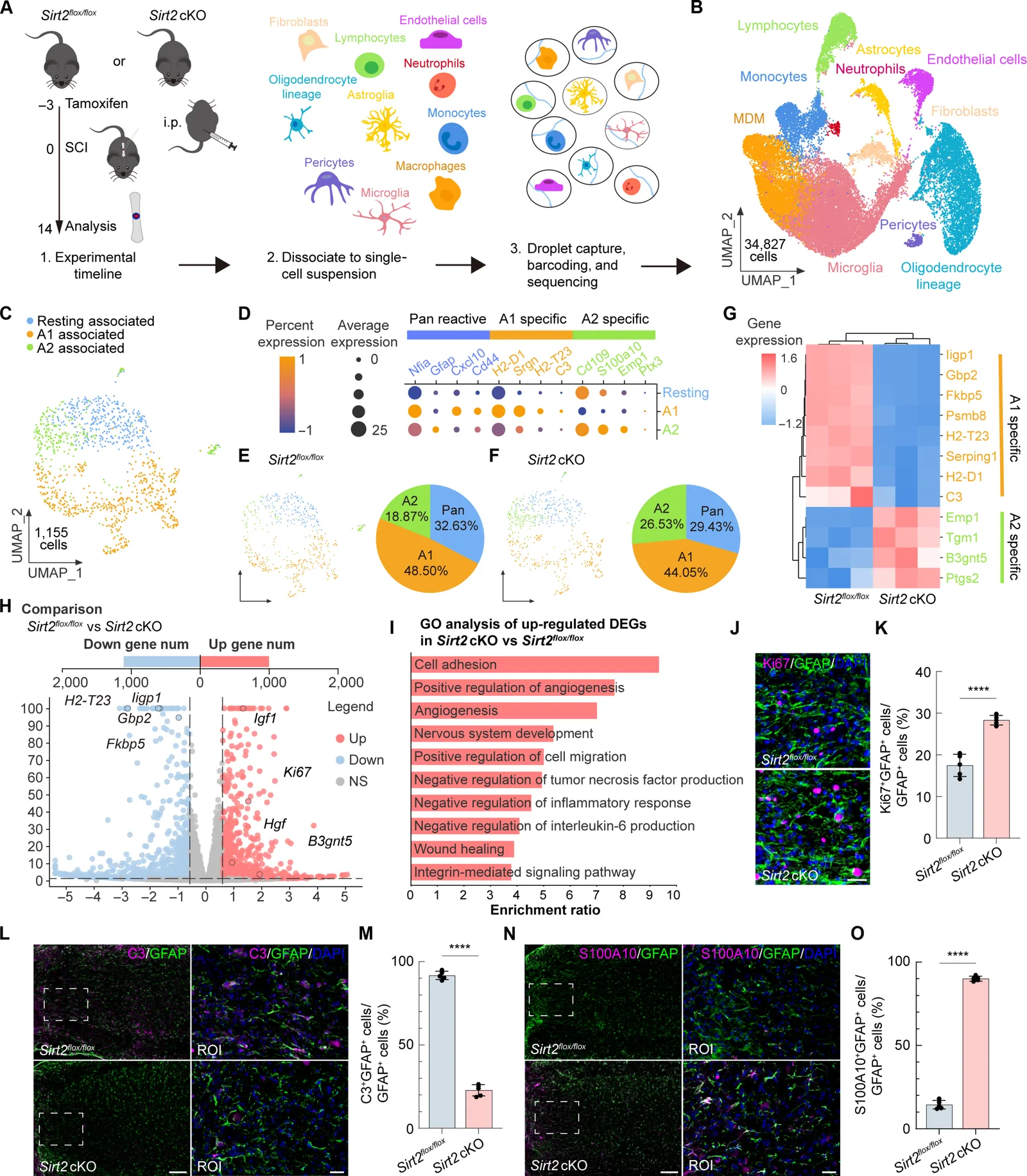
Astrocytic sirtuin2 (Sirt2) deficiency attenuates inflammatory astrocyte programs and enhances repair-associated features. (A) Single-cell RNA sequencing (scRNA-seq) workflow for injured spinal cords from control and *Sirt2* conditional knockout (cKO) mice at 14 d post-injury (dpi). (B) Uniform manifold approximation and projection (UMAP) of major cell populations. (C and D) Astrocyte reclustering into groups enriched for resting-associated, A1-associated, and A2-associated marker programs. (E and F) Relative proportions of the transcriptionally defined astrocyte groups in control and *Sirt2* cKO samples. (G and H) Bulk RNA sequencing (RNA-seq) of MACS-purified astrocytes showing A1-associated and A2-associated markers and representative differentially expressed genes. (I) Gene Ontology (GO) analysis of genes up-regulated in magnetic-activated cell sorting (MACS)-purified *Sirt2* cKO astrocytes by bulk RNA-seq. (J and K) Ki67 (purple), glial fibrillary acidic protein (GFAP; green), and 4′,6-diamidino-2-phenylindole (DAPI; blue) staining and quantification at 14 dpi. Scale bar, 20 μm. (L and M) C3 (purple), GFAP (green), and DAPI (blue) staining and quantification. Scale bars, 100 μm for overview images and 20 μm for insets. (N and O) S100A10 (purple), GFAP (green), and DAPI (blue) staining and quantification. Scale bars, 100 μm for overview images and 20 μm for insets. Data are mean ± standard error of the mean (SEM); *n* = 5 for (K), (M), and (O). For scRNA-seq, 8 mice per group were pooled. A 2-tailed unpaired Student *t* test was used for (K), (M), and (O). ****P < 0.0001.

We then examined astrocytes in greater detail. Reclustering separated astrocytes into 3 transcriptional groups enriched for resting-associated, A1-associated, or A2-associated marker programs (Fig. 3C and D) [57]. The group enriched for A1-associated markers expressed *H2-D1*, *Srgn*, *H2-T23*, *C3*, and related markers, whereas the group enriched for A2-associated markers expressed *Cd109*, *S100a10*, *Emp1*, *Ptgs2*, and other repair-associated genes [58]. Relative to controls, *Sirt2* cKO samples contained a lower proportion of astrocytes enriched for A1-associated markers and a higher proportion enriched for A2-associated markers (Fig. 3E and F). Differential gene-expression analysis further supported this broader transcriptional change: *Sirt2*-deficient astrocytes up-regulated repair-, metabolism-, and proliferation-associated genes such as *Cd14*, *Apoe*, and *Sox4* while down-regulating inflammatory genes such as *Stat1*, *Gbp2*, and *Ccl5* (Fig. S5D to F) [58–63]. Gene Ontology (GO) and KEGG enrichment of the genes increased in *Sirt2* cKO astrocytes revealed anti-inflammatory, neuroprotective, and tissue-repair programs, including reduced pro-inflammatory cytokine production, decreased neuronal apoptosis, axon extension, mitophagy, and oxidative phosphorylation (Fig. S5G and H). Thus, *Sirt2* deletion is associated with reduced inflammatory programs and enhanced repair-associated transcriptional features in astrocytes.

Because scRNA-seq may miss low-abundance transcripts and can be affected by sequencing depth, we validated the astrocyte findings using bulk RNA sequencing (RNA-seq) of magnetic-activated cell sorting (MACS)-purified astrocytes from injured spinal cords [64]. This independent dataset showed a pattern consistent with the single-cell results. A1-associated markers (*Psmb8*, *Serping1*, *H2-D1*, and *C3*) were reduced, whereas A2-associated markers (*Emp1*, *Tgm1*, *B3gnt5*, and *Ptgs2*) were increased in *Sirt2* cKO astrocytes (Fig. 3G) [58]. Bulk RNA-seq also showed higher expression of repair-associated genes, including *Igf1* and *Hgf*, as well as the proliferation marker *Ki67* (Fig. 3H). GO and KEGG enrichment analyses identified pathways related to cell adhesion, angiogenesis, nervous system development, axon guidance, wound healing, and suppression of inflammatory cytokine responses (Fig. 3I and Fig. S5I). These data further suggest that *Sirt2* deletion enhances proliferative and repair-associated transcriptional programs after SCI.

In situ immunofluorescence provided histological support for the sequencing results. GFAP^+^Ki67^+^ proliferating astrocytes were more abundant in *Sirt2* cKO spinal cords (Fig. 3J and K). Consistent with the transcriptomic profiles, GFAP^+^C3^+^ astrocytes were decreased, whereas GFAP^+^S100A10^+^ astrocytes were increased in the injured region of *Sirt2* cKO mice (Fig. 3L to O). To determine whether these reactive astrocyte marker changes were associated with lactate-related H4K8la accumulation, *Sirt2* cKO mice were treated with stiripentol, a lactate dehydrogenase inhibitor with established CNS bioavailability [65–67]. Stiripentol attenuated the increase in astrocytic H4K8la induced by *Sirt2* deletion and reduced the associated changes in GFAP^+^C3^+^ and GFAP^+^S100A10^+^ astrocytes (Fig. S6A to C). Together, these results suggest that astrocyte-specific *Sirt2* deletion attenuates A1-associated inflammatory features while enhancing A2-associated and repair-associated features, thereby generating a lesion environment more permissive for recovery.

### Astrocytic Sirt2 deletion reduces pathological vascular remodeling and improves BSCB integrity

Because astrocytes and endothelial cells interact closely within the neurovascular unit [68], we asked whether the loss of *Sirt2* in astrocytes altered endothelial responses after SCI. Cell–cell communication analysis of the scRNA-seq dataset identified extensive predicted ligand–receptor interactions between astrocytes and endothelial cells, indicating strong intercellular coupling after SCI (Fig. S7A). Endothelial reclustering resolved 4 states: homeostatic, differentiating, neovascularizing, and perivascular macrophage-associated endothelial cells (Fig. S7B and C). In control spinal cords, the perivascular macrophage-associated cluster predominated and homeostatic endothelial cells were relatively scarce. In contrast, *Sirt2* cKO increased the homeostatic endothelial fraction and decreased the perivascular macrophage-associated fraction (Fig. S7D and E), suggesting a shift toward a more stable endothelial state that supports barrier function.

We explored the molecular basis of the endothelial shift by comparing endothelial gene expression between control and *Sirt2* cKO mice (Fig. 4A and B). In *Sirt2* cKO mice, endothelial cells expressed higher levels of barrier-associated genes, including *Cldn5* and *Ocln* [69], together with increased CD31, while inflammatory genes such as *Ccl5*, *Cxcl10*, and *Ly86* were decreased (Fig. 4B and E). Enrichment analysis linked genes elevated in *Sirt2* cKO endothelial cells to angiogenesis, vasculogenesis, cell–cell adhesion, barrier formation, tight-junction assembly, and blood–brain barrier development (Fig. 4C). KEGG analysis highlighted tight junction, adherens junction, and gap junction pathways (Fig. 4D). These findings indicate that astrocytic *Sirt2* loss promotes an endothelial program compatible with BSCB reconstruction while reducing inflammatory and pathological vascular features.

**Fig. 4.**
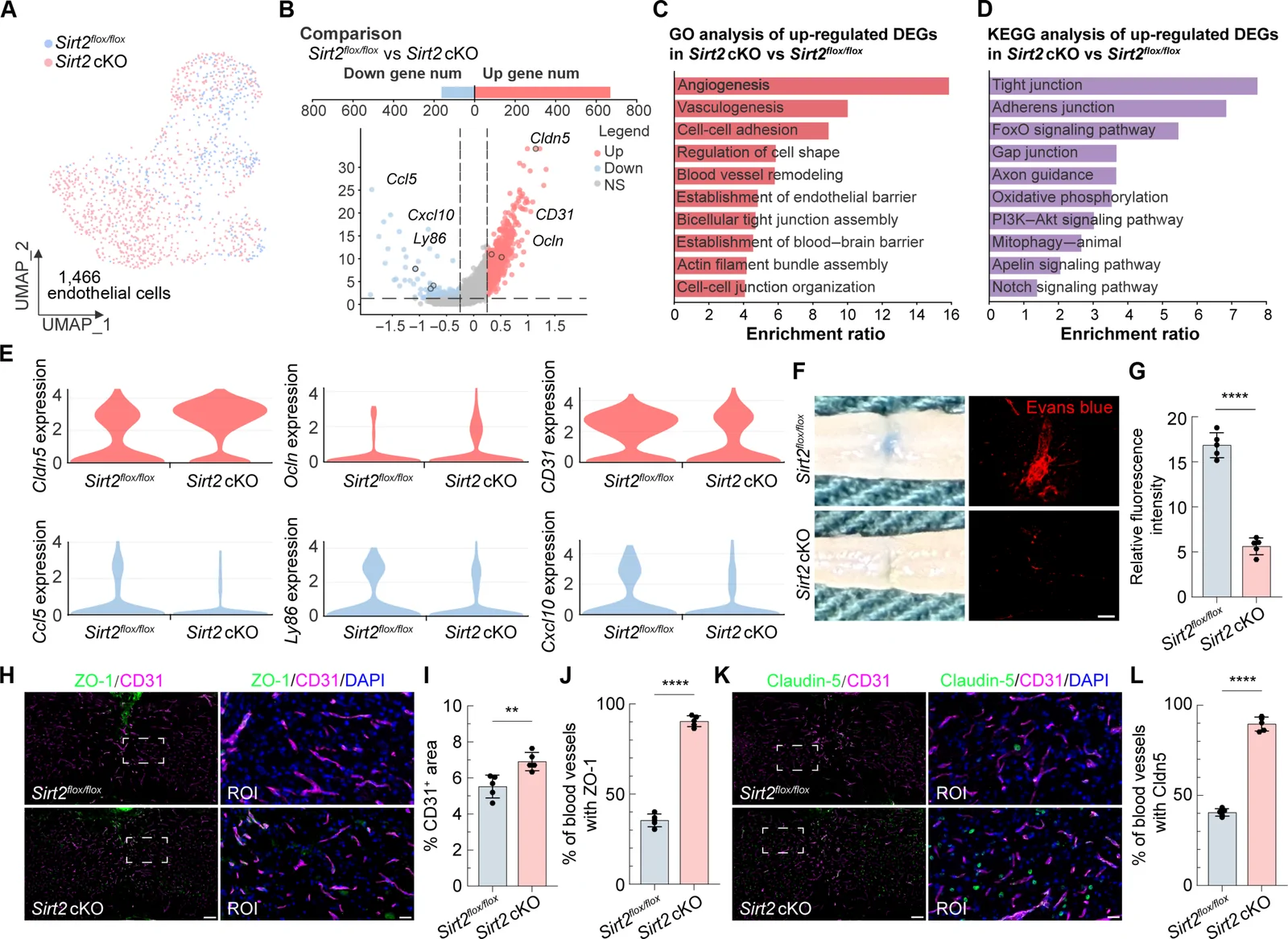
Astrocytic sirtuin2 (Sirt2) deletion reduces pathological vascular remodeling and improves blood–spinal cord barrier (BSCB) integrity. (A and B) Uniform manifold approximation and projection (UMAP) and volcano plot of endothelial gene-expression changes. (C and D) Gene Ontology (GO) and Kyoto Encyclopedia of Genes and Genomes (KEGG) enrichment of genes increased in *Sirt2* conditional knockout (cKO) endothelial cells. (E) Violin plots of selected endothelial-barrier- and inflammation-related genes. (F and G) Evans blue fluorescence and quantification at 14 d post-injury (dpi). Scale bar, 100 μm. (H to J) Zonula occludens-1 (ZO-1; green), CD31 (purple), and 4′,6-diamidino-2-phenylindole (DAPI; blue) staining, with quantification of CD31^+^ vascular area and ZO-1^+^ vessels. Scale bars, 200 μm for overview images and 20 μm for insets. (K and L) Claudin-5 (green), CD31 (purple), and DAPI (blue) staining, with quantification of claudin-5^+^ vessels. Scale bars, 200 μm for overview images and 20 μm for insets. Data are mean ± standard error of the mean (SEM); *n* = 5 for (G), (I), (J), and (L). A 2-tailed unpaired Student *t* test was used for (G), (I), (J), and (L). **P < 0.01; ****P < 0.0001.

We further tested whether these transcriptional changes translated into structural and functional BSCB improvement. Evans blue (EB) leakage was markedly lower in *Sirt2* cKO mice, indicating reduced vascular permeability after injury (Fig. 4F and G). Immunostaining showed greater vascular coverage of the tight-junction proteins zonula occludens-1 (ZO-1) and claudin-5 and higher proportions of ZO-1^+^ and claudin-5^+^ vessels in *Sirt2* cKO spinal cords (Fig. 4H and J to L). The CD31^+^ vascular area was also increased in *Sirt2* cKO mice (Fig. 4H and I). These results indicate that astrocytic *Sirt2* deletion promotes vessel preservation or reconstruction and improves functional BSCB integrity after SCI.

### Astrocytic Sirt2 deletion limits inflammation, preserves neurons, increases serotonergic fibers, and improves recovery

Because stronger vascular integrity should reduce inflammatory entry and secondary tissue damage [12,13,70], we examined inflammatory cell accumulation, neuronal preservation, descending serotonergic fibers, and locomotor behavior. In control injured spinal cords, CD68^+^ inflammatory cells accumulated broadly, especially around the lesion center. This accumulation was substantially reduced in *Sirt2* cKO mice, most notably in the Z1, Z2, and Z3 regions (Fig. 5A and B). Thus, loss of *Sirt2* in astrocytes limits local inflammatory cell accumulation after SCI.

**Fig. 5.**
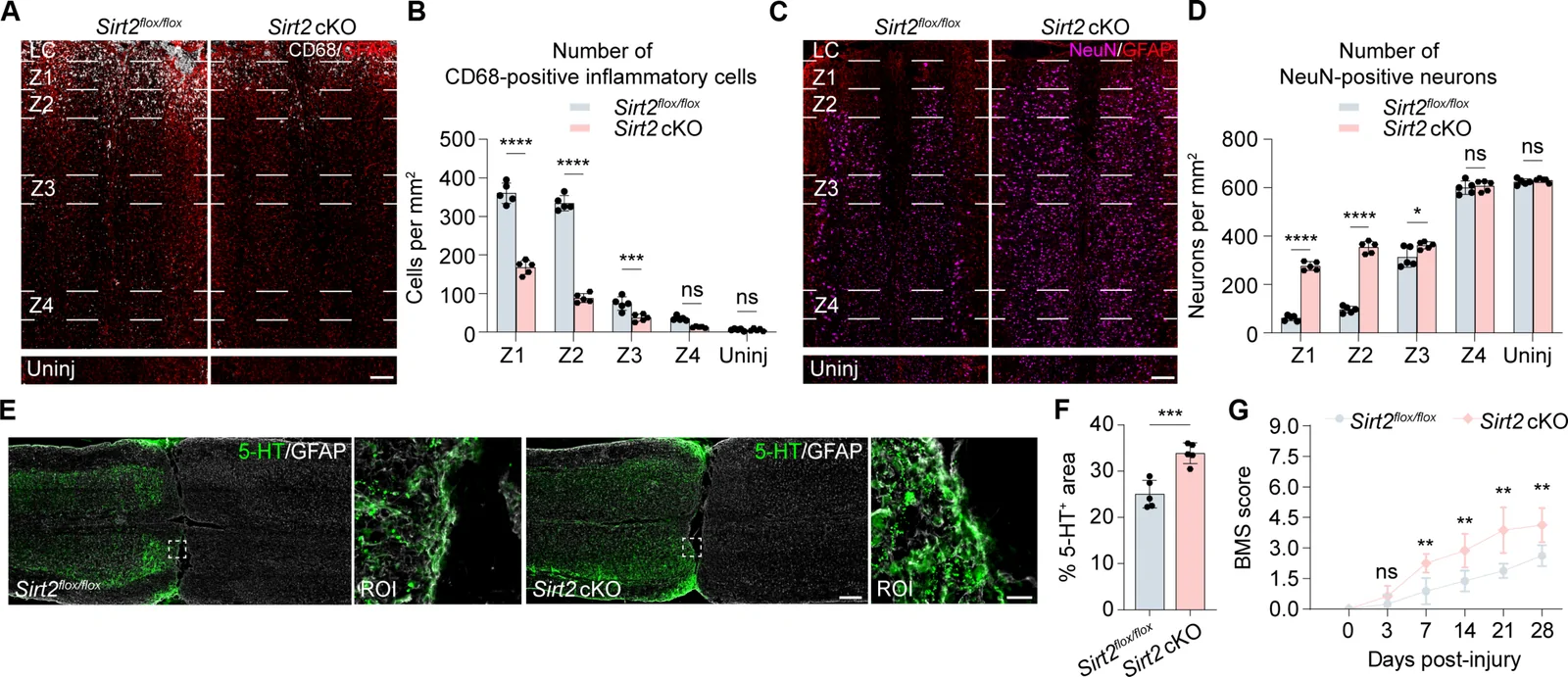
Astrocytic sirtuin2 (Sirt2) deletion reduces inflammation, preserves neurons, increases 5-hydroxytryptamine (5-HT) fibers, and improves recovery. (A and B) CD68 (white) and glial fibrillary acidic protein (GFAP; red) staining and quantification in horizontal spinal cord sections at 28 d post-injury (dpi). LC, lesion core; Z1, 0 to 250 μm; Z2, 250 to 500 μm; Z3, 1,000 to 1,250 μm; Z4, 2,000 to 2,250 μm; Uninj, uninjured region. Scale bar, 200 μm. (C and D) NeuN (purple) and GFAP (red) staining and quantification of NeuN^+^ neurons in the indicated regions. Scale bar, 200 μm. (E and F) 5-HT (green) and GFAP (white) staining and quantification of 5-HT^+^ fiber area. Scale bars, 200 μm for overview images and 20 μm for insets. (G) Basso Mouse Scale (BMS) scores after spinal cord injury (SCI). Data are mean ± standard error of the mean (SEM); *n* = 5 for (B), (D), and (F); *n* = 8 for (G). Two-way analysis of variance (ANOVA) with Tukey’s test was used for (B), (D), and (G); a 2-tailed unpaired Student *t* test was used for (F). ns, not significant; *P < 0.05; **P < 0.01; ***P < 0.001; ****P < 0.0001.

We then evaluated neuronal survival using NeuN staining. Relative to controls, *Sirt2* cKO mice retained more NeuN^+^ neurons in the injured spinal cord, with statistically significant increases in Z1, Z2, and Z3 but no detectable difference in Z4 or uninjured tissue (Fig. 5C and D). Because serotonergic projections contribute to locomotor recovery after SCI, we also quantified 5-hydroxytryptamine (5-HT)-positive fibers [71]. *Sirt2* cKO mice showed higher serotonergic fiber density than controls (Fig. 5E and F). In line with these histological improvements, Basso Mouse Scale (BMS) scores were higher in *Sirt2* cKO mice from 7 dpi onward (Fig. 5G). Together, these findings show that astrocytic *Sirt2* deletion reduces inflammation, preserves neural tissue, increases serotonergic fiber density, and enhances locomotor recovery after SCI.

### Sirt2 pharmacological inhibition mimics the benefits of astrocytic Sirt2 deletion after SCI

We next tested whether pharmacological Sirt2 inhibition could reproduce the effects of genetic deletion. SCI mice were treated with AK-7, a functional inhibitor of Sirt2 (Fig. 6A) [72]. AK-7 increased astrocytic Pan-Kla and H4K8la levels in GFAP^+^ cells, indicating the suppression of astrocytic delactylase activity in vivo (Fig. 6B to D).

**Fig. 6.**
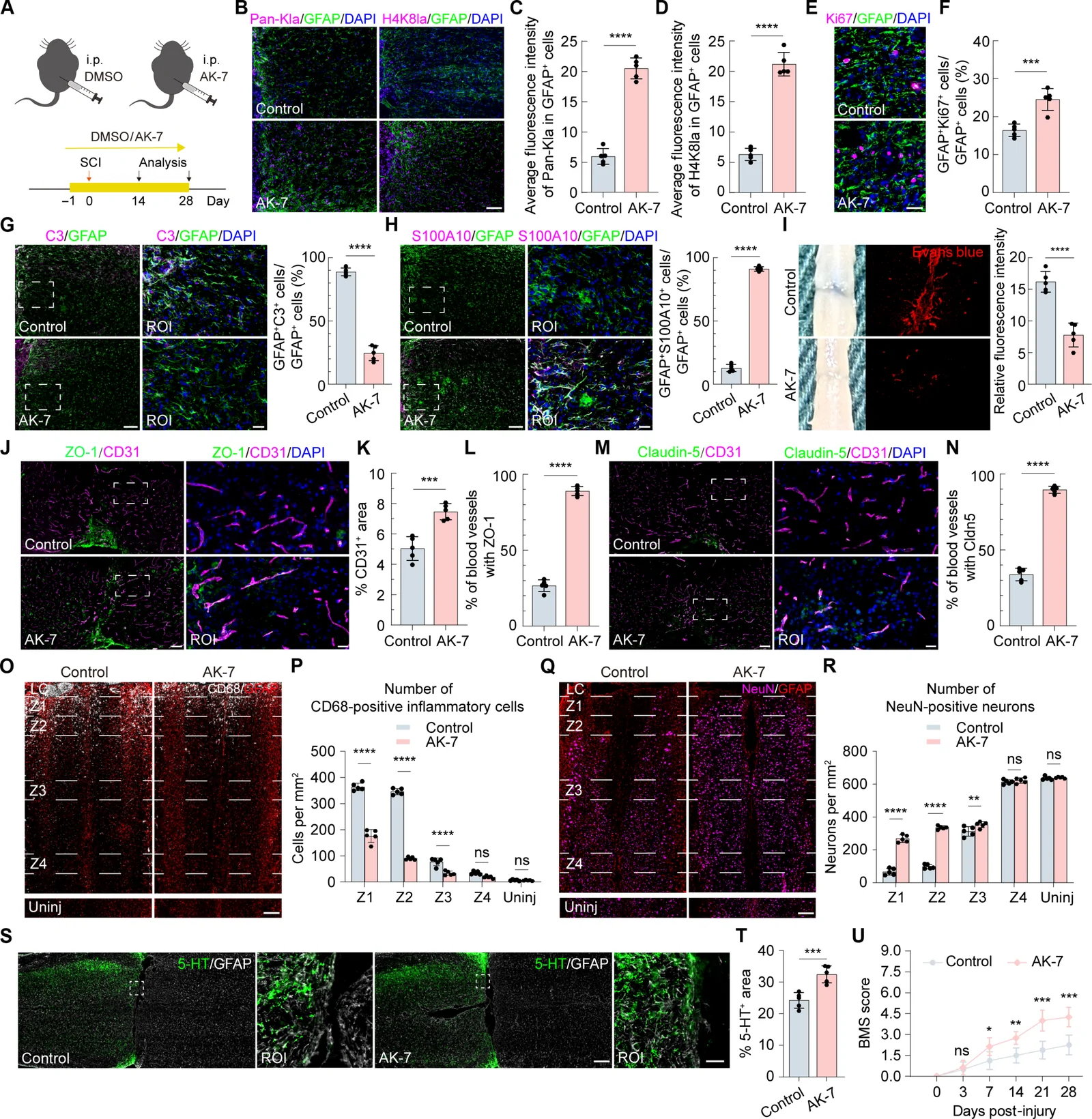
AK-7 mimics the protective effects of astrocytic sirtuin2 (Sirt2) deletion after spinal cord injury (SCI). (A) AK-7 treatment schedule. (B to D) Pan-Kla (purple), glial fibrillary acidic protein (GFAP; green), H4K8la (red), and 4′,6-diamidino-2-phenylindole (DAPI; blue) staining, with quantification in vehicle- and AK-7-treated mice at 14 d post-injury (dpi). Scale bar, 50 μm. (E and F) Ki67 (purple), GFAP (green), and DAPI (blue) staining and quantification. Scale bar, 20 μm. (G) C3 (purple), GFAP (green), and DAPI (blue) staining and quantification. Scale bars, 100 μm for overview images and 20 μm for insets. (H) S100A10 (purple), GFAP (green), and DAPI (blue) staining and quantification. Scale bars, 100 μm for overview images and 20 μm for insets. (I) Evans blue fluorescence and quantification. Scale bar, 100 μm. (J to L) Zonula occludens-1 (ZO-1; green), CD31 (purple), and DAPI (blue) staining, with quantification of CD31^+^ vascular area and ZO-1^+^ vessels. Scale bars, 200 μm for overview images and 20 μm for insets. (M and N) Claudin-5 (green), CD31 (purple), and DAPI (blue) staining, with quantification of claudin-5^+^ vessels. Scale bars, 200 μm for overview images and 20 μm for insets. (O and P) CD68 (white) and GFAP (red) staining with quantification of CD68^+^ inflammatory cells at 28 dpi. Scale bar, 200 μm. (Q and R) NeuN (purple) and GFAP (red) staining with quantification of NeuN^+^ neurons at 28 dpi. Scale bar, 200 μm. (S and T) 5-Hydroxytryptamine (5-HT; green) staining and quantification of 5-HT^+^ fibers at 28 dpi. Scale bars, 200 μm for overview images and 20 μm for insets. (U) Basso Mouse Scale (BMS) scores after SCI. Data are mean ± standard error of the mean (SEM); *n* = 5 for image quantification and Evans blue analysis; *n* = 8 for (U). A two-tailed unpaired Student *t* test was used for (C), (D), (F) to (I), (K), (L), (N), and (T), and 2-way analysis of variance (ANOVA) with Tukey’s test was used for (P), (R), and (U). ns, not significant; *P < 0.05; **P < 0.01; ***P < 0.001; ****P < 0.0001.

AK-7 also altered reactive astrocyte features after SCI. GFAP^+^Ki67^+^ proliferating astrocytes increased after AK-7 treatment (Fig. 6E and F), while GFAP^+^C3^+^ astrocytes decreased and GFAP^+^S100A10^+^ astrocytes increased (Fig. 6G and H). These marker changes suggest that AK-7 treatment attenuates inflammatory astrocyte features while enhancing repair-associated features, consistent with genetic *Sirt2* deletion.

We then examined vascular integrity and remodeling after AK-7 treatment. EB leakage was reduced in AK-7-treated mice, consistent with improved BSCB function (Fig. 6I). ZO-1 and claudin-5 coverage of CD31^+^ vessels increased, and CD31^+^ vascular area was higher after AK-7 administration (Fig. 6J to N). These findings indicate that pharmacological Sirt2 inhibition suppresses pathological vascular remodeling and promotes BSCB reconstruction after SCI. AK-7 also reduced CD68^+^ inflammatory cell accumulation, preserved NeuN^+^ neurons in Z1 to Z3, increased 5-HT fiber density, and improved BMS scores from 7 dpi onward (Fig. 6O to U). Overall, AK-7 reproduced the major benefits of astrocyte-specific *Sirt2* deletion, including enhanced astrocytic lactylation, enhanced repair-associated astrocyte features, improved BSCB repair, reduced inflammation, neuronal preservation, increased serotonergic fiber density, and improved locomotor recovery.

### H4K8la activates Hgf transcription in astrocytes lacking Sirt2 after SCI

To define how *Sirt2*-mediated delactylation regulates astrocyte function at the epigenetic level after SCI, we performed H4K8la CUT&Tag in astrocytes isolated from control and *Sirt2* cKO mice at 14 dpi. Compared with controls, *Sirt2* cKO astrocytes showed higher H4K8la peak enrichment around transcription start sites (Fig. 7A). Genome-wide mapping showed that most H4K8la peaks were located within gene promoters (Fig. 7B), consistent with the role of H4K8la as an activating chromatin mark. KEGG enrichment highlighted protein processing in the endoplasmic reticulum, ubiquitin-mediated proteolysis, and autophagy, whereas GO analysis identified processes related to mitotic cell cycle regulation and proteasome-mediated protein catabolism (Fig. S8A and B). These results indicate that *Sirt2* cKO-driven H4K8la accumulation is associated with broad transcriptional remodeling of astrocytic programs related to proteostasis, metabolism, and cell cycle control.

**Fig. 7.**
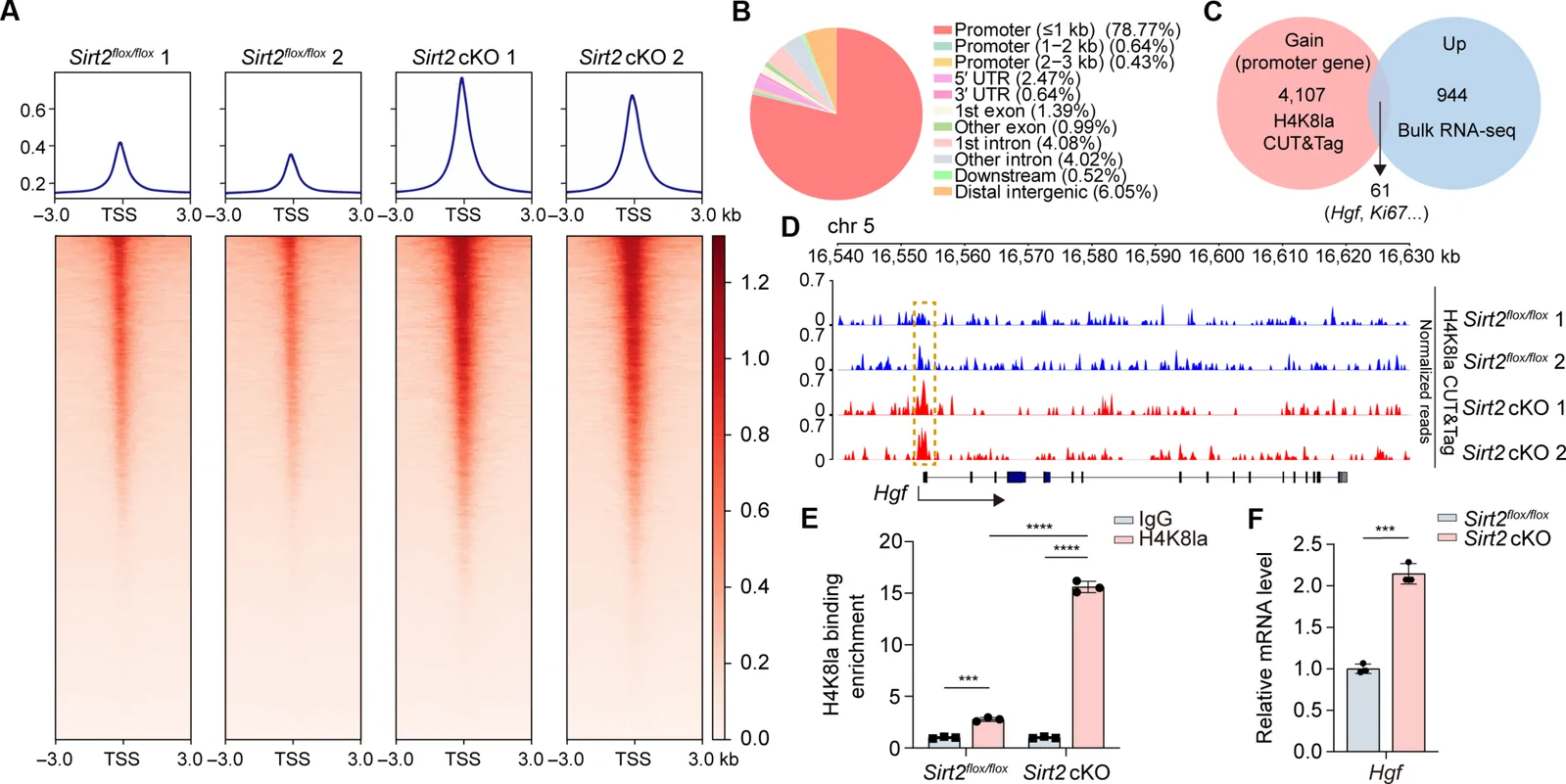
H4K8la is associated with hepatocyte growth factor (Hgf) transcriptional activation in sirtuin2 (Sirt2)-deficient astrocytes. (A) H4K8la CUT&Tag profiles around transcription start sites in purified astrocytes from control and *Sirt2* conditional knockout (cKO) mice at 14 d post-injury (dpi). (B) Genomic distribution of H4K8la peaks in *Sirt2* cKO astrocytes. (C) Integrated H4K8la CUT&Tag and bulk RNA sequencing (RNA-seq) analysis identifying genes with increased promoter H4K8la and elevated expression. (D) Genome-browser tracks showing H4K8la enrichment at the *Hgf* promoter. (E) Chromatin immunoprecipitation–quantitative polymerase chain reaction (ChIP–qPCR) validation of H4K8la enrichment at the *Hgf* promoter; immunoglobulin G (IgG) was used as a negative control. (F) qPCR analysis of *Hgf* messenger RNA (mRNA) in purified astrocytes. Data are mean ± standard error of the mean (SEM); *n* = 2 for (A) to (D); *n* = 3 for (E) and (F). A 2-tailed unpaired Student *t* test was used for (E) and (F). ***P < 0.001; ****P < 0.0001.

To identify candidate downstream genes, we integrated CUT&Tag peaks with increased promoter H4K8la and bulk RNA-seq genes up-regulated in *Sirt2* cKO astrocytes. This analysis identified 61 overlapping genes with both higher promoter H4K8la and higher expression in *Sirt2* cKO astrocytes, including *Hgf* and *Ki67* (Fig. 7C). These results suggest that increased H4K8la may contribute to the transcriptional activation of multiple targets. Among them, HGF was particularly notable because of its established roles in angiogenesis, endothelial protection, and tissue repair [73–77]. Genome-browser visualization showed enhanced H4K8la occupancy at the *Hgf* promoter, and chromatin immunoprecipitation–quantitative polymerase chain reaction (ChIP–qPCR) confirmed increased H4K8la enrichment at this locus in *Sirt2* cKO astrocytes (Fig. 7D and E). qPCR further showed increased *Hgf* messenger RNA (mRNA) in *Sirt2* cKO astrocytes (Fig. 7F). Together, these results support an association between increased H4K8la enrichment at the *Hgf* promoter and elevated *Hgf* expression following astrocytic *Sirt2* deletion.

### HGF is required for the repair-promoting effects of astrocytic Sirt2 deletion after SCI

To determine whether *Hgf* is necessary for the protective effects of astrocytic *Sirt2* deletion, we used adeno-associated virus (AAV)-delivered short hairpin RNA (shRNA) to reduce *Hgf* in astrocytes of *Sirt2* cKO mice (Fig. 8A and B). Immunofluorescence confirmed efficient astrocyte-targeted viral transduction and showed that HGF staining was reduced after sh*Hgf* treatment (Fig. S9A and B). These results verified effective *Hgf* knockdown in vivo.

**Fig. 8.**
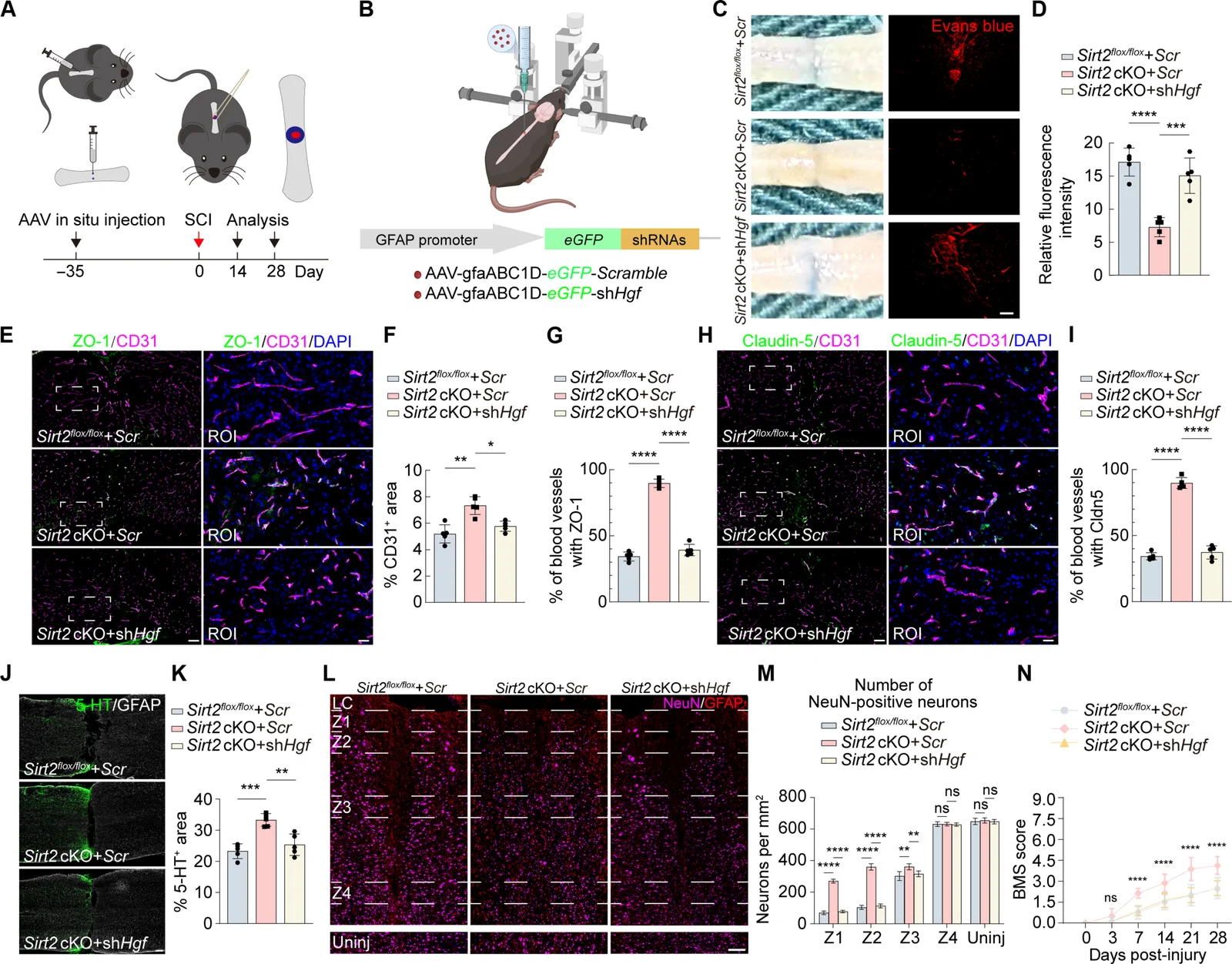
Hepatocyte growth factor (HGF) is required for the reparative effects driven by astrocytic sirtuin2 (Sirt2) deletion after spinal cord injury (SCI). (A and B) Experimental strategy and adeno-associated virus (AAV) design for astrocytic *Hgf* knockdown in *Sirt2* conditional knockout (cKO) mice. (C and D) Evans blue fluorescence and quantification in *Sirt2^flox/flox^* + Scramble, *Sirt2* cKO + Scramble, and *Sirt2* cKO + sh*Hgf* mice at 14 d post-injury (dpi). Scale bar, 100 μm. (E to G) Zonula occludens-1 (ZO-1; green), CD31 (purple), and 4′,6-diamidino-2-phenylindole (DAPI; blue) staining, with quantification of CD31^+^ vascular area and ZO-1^+^ vessels at 14 dpi. Scale bars, 200 μm for overview images and 20 μm for insets. (H and I) Claudin-5 (green), CD31 (purple), and DAPI (blue) staining, with quantification of claudin-5^+^ vessels at 14 dpi. Scale bars, 200 μm for overview images and 20 μm for insets. (J and K) 5-Hydroxytryptamine (5-HT; green) staining and quantification of 5-HT^+^ fibers at 28 dpi. Scale bar, 200 μm. (L and M) NeuN (purple) and glial fibrillary acidic protein (GFAP; red) staining with quantification of NeuN^+^ neurons in the indicated regions at 28 dpi. Scale bar, 200 μm. (N) Basso Mouse Scale (BMS) scores after SCI. Data are mean ± standard error of the mean (SEM); *n* = 5 for (D), (F), (G), (I), (K), and (M); *n* = 8 for N. One-way analysis of variance (ANOVA) with Tukey’s test was used for (D), (F), (G), (I), and (K); 2-way ANOVA with Tukey’s test was used for (M) and (N). ns, not significant; *P < 0.05, **P < 0.01, ***P < 0.001, ****P < 0.0001.

We next evaluated BSCB integrity and vascular remodeling. *Hgf* knockdown increased EB leakage, indicating loss of the barrier-protective effect produced by *Sirt2* deletion (Fig. 8C and D). *Hgf* knockdown also reduced the proportions of ZO-1^+^ and claudin-5^+^ vessels and decreased CD31^+^ vascular area (Fig. 8E to I). Therefore, HGF is necessary for the BSCB reconstruction and suppression of pathological vascular remodeling induced by *Sirt2* deletion. *Hgf* knockdown also worsened outcomes in *Sirt2* cKO mice: NeuN^+^ neuron preservation declined, 5-HT fiber density decreased, and locomotor recovery fell (Fig. 8J to N). These findings demonstrate that HGF functions downstream of astrocytic *Sirt2* deletion to support BSCB reconstruction, preserve neurons, increase serotonergic fiber density, and promote recovery.

### Astrocytic Sirt2 deletion attenuates CD8^+^ T-cell-related inflammatory responses after SCI

Previous work has shown that astrocytes can recruit peripheral immune cells into injured CNS tissue and intensify secondary damage [78–80]. Recent findings also implicate astrocyte-dependent CD8^+^ T-cell recruitment in neural injury [14,81,82]. We therefore analyzed whether astrocytic *Sirt2* deletion affected lymphocyte remodeling after SCI. Reclustering of lymphocytes identified CD4^+^ T cells, CD8^+^ T cells, and B cells according to canonical markers, including *Cd4*, *Cd3d*, *Cd8a*, *Cd8b1*, *Ly6d*, *Cd79a*, and *Cd79b* (Fig. S10A and B). Compared with controls, *Sirt2* cKO spinal cords showed a clear reduction in CD8^+^ T cells and an increase in CD4^+^ T cells, whereas B-cell abundance changed little (Fig. S10C and D). Ligand–receptor analysis revealed astrocyte–CD8^+^ T cell communication pairs, including Cd55–Adgre5, Fas–Fasl, Cntn1–Notch2, Sema4d–Ptprc, and several collagen–integrin interactions (Fig. S10E). These interactions were markedly weaker in *Sirt2* cKO mice, suggesting that astrocyte–CD8^+^ T cell communication is attenuated after astrocytic *Sirt2* loss.

Consistent with this cellular shift, lymphocyte differential expression analysis showed lower levels of cytotoxic effector genes, including *Gzma*, *Gzmb*, and *Nkg7*, in *Sirt2* cKO mice, suggesting reduced cytotoxic lymphocyte activity [81,83]. In contrast, genes associated with immunomodulatory lymphocyte states, including *Il2rb*, *Gata3*, and *Ikzf2*, were increased (Fig. S10F and G), indicating a shift toward a less inflammatory lymphocyte profile [84–87]. Functional enrichment of down-regulated genes further highlighted T-cell receptor signaling, T-cell activation, chemokine signaling, natural-killer-cell-mediated cytotoxicity, apoptosis, and necroptosis (Fig. S10H and I). Together, these findings indicate that astrocyte-specific *Sirt2* deletion restrains CD8^+^ T-cell-related inflammatory responses and may protect injured tissue by reducing pathogenic lymphocyte accumulation or cytotoxic activity.

## Discussion

BSCB repair is increasingly recognized as essential for neurological recovery after SCI, yet the intrinsic mechanisms governing effective barrier repair remain poorly defined [5,6,9,10,12,13]. Here, we identify astrocytic delactylation as a previously unrecognized mechanism that restrains BSCB repair after injury. We show that Sirt2 is induced in astrocytes within the lesion border and closely associated with chronic pathological vascular remodeling. Astrocyte-specific *Sirt2* deletion enhanced BSCB repair and locomotor recovery; these effects were largely reproduced by pharmacological Sirt2 inhibition, whereas several were attenuated by *Hgf* knockdown. Collectively, these findings define an astrocytic *Sirt2*–H4K8la–*Hgf* axis that connects metabolic state, epigenetic regulation, and BSCB reconstruction after SCI (Fig. S11).

A key conclusion is that astrocytic Sirt2 is tightly linked to persistent vascular pathology after SCI. Although astrocytes are known regulators of endothelial function in injured CNS tissue, their influence on BSCB repair has remained debated [18–22]. In our study, Sirt2 was the most enriched delactylase in astrocytes after SCI, and its expression peaked when microvascular dilation was greatest. The parallel temporal pattern, together with the positive correlation between astrocytic Sirt2 and vessel diameter, supports an association between astrocytic Sirt2 expression and microvascular dilation and is consistent with a potential contribution of increased astrocytic delactylation to barrier failure and pathological vascular remodeling. These data extend current understanding of astrocyte–vascular interactions by identifying delactylation as an additional regulatory layer beyond canonical cytokine signaling.

Our findings suggest that *Sirt2* suppresses astrocytic lactylation through 2 cooperating mechanisms. First, *Sirt2* directly limits global lysine lactylation and H4K8la levels through delactylase activity. Second, loss of *Sirt2* changes astrocyte metabolism by increasing glycolytic output, lactate accumulation, and mitochondrial respiration, thereby generating metabolic conditions that favor lactylation. The simultaneous increases in glycolysis and respiration likely reflect enhanced overall metabolic activity and flexibility, consistent with the increased abundance of GFAP^+^Ki67^+^ astrocytes, rather than a complete metabolic shift toward glycolysis. However, mitochondrial abundance and substrate utilization were not directly assessed. This model places *Sirt2* at the interface of metabolism and epigenetic regulation, allowing injury-induced metabolic remodeling to be converted into sustained transcriptional responses through lactylation-dependent mechanisms.

Another important implication is that inhibiting astrocytic delactylation is associated with a reactive astrocyte profile characterized by enhanced repair-associated features. Reactive astrocytes are heterogeneous and can exert harmful or beneficial effects in the injured CNS [57,58]. Transcriptomic and in situ analyses consistently indicated that *Sirt2* deletion reduced inflammatory programs associated with A1 markers while enhancing programs associated with A2 markers and tissue repair. The A1/A2 framework is a simplified descriptor rather than a definition of discrete astrocyte populations, and C3, S100A10, and related gene sets capture only selected features of the continuous and context-dependent astrocyte responses after SCI. These data suggest that astrocytic delactylation modulates selected reactive astrocyte programs after injury and may influence inflammatory and repair-supportive responses. Because astrocytes coordinate multiple features of the lesion microenvironment, these Sirt2-associated transcriptional changes may contribute to improved vascular stability, reduced inflammation, and enhanced neuroprotection. The coordinated vascular, inflammatory, neural, and behavioral improvements support a model in which astrocytic delactylation contributes to endothelial barrier failure and chronic vascular pathology after SCI. Thus, inhibiting astrocytic delactylation may support recovery through coordinated benefits affecting the vasculature, inflammatory milieu, and neural circuits. Although increased 5-HT-positive fiber density was detected only rostral to the lesion, spared serotonergic fibers may contribute to locomotor recovery by reorganizing relay circuits through rostrally spared neurons, a possibility that requires further validation [4,88].

The translational relevance of Sirt2 targeting is an important aspect of this study. Unlike genetic manipulation, pharmacological inhibition with AK-7 reproduced the major protective effects of astrocyte-specific *Sirt2* deletion, including greater astrocytic lactylation, reduced pathological vascular remodeling, restored BSCB integrity, attenuated inflammation, and improved locomotor performance. The agreement between genetic and pharmacological approaches indicates that Sirt2 is both mechanistically important and therapeutically actionable. Because BSCB disruption, chronic vascular leakage, and secondary inflammation are major pathological features of human SCI [10,14], astrocytic Sirt2 inhibition may provide a strategy to target multiple damaging processes simultaneously. More broadly, these results support the concept that astrocyte metabolic and epigenetic regulators can be harnessed for neurovascular repair.

Mechanistically, our data identify *Hgf* as a key downstream effector of *Sirt2* deficiency. CUT&Tag showed that *Sirt2* loss preferentially increased H4K8la at promoter regions, consistent with the activating role of this chromatin mark. Integrating H4K8la occupancy with transcriptomic data identified genes with both increased promoter H4K8la and increased expression. HGF emerged as a particularly compelling candidate because of its established functions in angiogenesis, endothelial protection, and tissue repair [73–77]. Higher H4K8la enrichment at the *Hgf* promoter, together with elevated *Hgf* mRNA, supports an association between promoter-associated H4K8la and enhanced *Hgf* expression and is consistent with H4K8la-linked epigenetic regulation of *Hgf* in the context of reduced Sirt2-dependent delactylation. Functionally, *Hgf* knockdown markedly attenuated the effects of *Sirt2* deficiency on BSCB integrity, pathological vascular remodeling, neuronal preservation, serotonergic fiber density, and locomotor recovery, demonstrating that *Hgf* is required for the reparative consequences of *Sirt2* inhibition. *Hgf* may not be the only astrocyte-derived mediator of the vascular and neural effects of *Sirt2* deletion. VEGF-A and other astrocyte-derived paracrine factors may also contribute, but their roles and relationship with *Hgf* after SCI require further investigation [89,90].

Beyond vascular and astrocytic mechanisms, our findings indicate that astrocytic *Sirt2* deletion alters the lymphocyte compartment after SCI. *Sirt2* cKO spinal cords contained fewer CD8^+^ T cells, weaker astrocyte–CD8^+^ T cell communication signatures, and reduced cytotoxic lymphocyte programs. These observations are notable because recent evidence suggests that recruited CD8^+^ T cells can promote endothelial damage and neuronal loss after SCI [14,81,82,91]. Therefore, *Sirt2* deficiency may protect injured tissue not only by improving BSCB reconstruction and astrocyte state but also by limiting pathogenic CD8^+^ T-cell accumulation or persistence, extending the influence of astrocytic delactylation to the peripheral immune components of secondary injury.

This study has several limitations. First, the upstream signals inducing Sirt2 after injury remain unknown, and additional genes beyond Hgf may contribute to the reparative phenotype. Second, because an independent Cre reporter line was not used, the proportion and lineage distribution of recombined cells could not be determined, and ectopic recombination in progenitor or other cell populations cannot be excluded. Third, pooling 8 mice per group for scRNA-seq prevented assessment of interanimal variability; thus, these findings should be considered exploratory. Fourth, multiple-testing correction was not applied to the untargeted metabolomic analysis; therefore, these metabolomic findings should also be interpreted as exploratory. Fifth, systemic AK-7 is not astrocyte specific, and its pharmacokinetics and penetration across the disrupted BSCB were not directly assessed. Sixth, increased 5-HT-positive fiber density cannot distinguish preservation or local sprouting from long-distance regeneration, and corticospinal tract tracing was not performed. Seventh, behavioral assessment was limited to locomotor recovery and did not evaluate sensory abnormalities or pain. Finally, all experiments were conducted in mice, and the relevance of these findings to human SCI remains uncertain.

In conclusion, this study establishes a mechanistic framework linking astrocyte metabolism, histone lactylation, and BSCB reconstruction after SCI. By defining astrocytic Sirt2 as a negative regulator of BSCB repair and identifying the H4K8la–*Hgf* axis as a downstream pathway, we reveal a previously unappreciated mode of neurovascular regulation in the injured spinal cord. The ability of pharmacological Sirt2 inhibition to reproduce the benefits of astrocyte-specific *Sirt2* deletion highlights the translational potential of this pathway. Overall, these findings suggest that inhibiting astrocytic delactylation, particularly through targeting Sirt2, may promote BSCB reconstruction, limit secondary injury, and improve recovery after SCI.

## Materials and Methods

### Experimental animals

C57BL/6 wild-type (WT) mice of both sexes were used at 8 to 12 weeks of age (20 to 25 g) and were obtained from the Laboratory Animal Center of Anhui Medical University. The Animal Committee of Anhui Medical University approved all procedures under protocol LLSC20220381. CCR2^RFP^CX3CR1^GFP^ reporter mice (8 weeks old; Catalog No. 032127) were purchased from Shanghai Model Organism. *Aldh1l1^CreERT2^*-mediated *Sirt2* cKO mice were generated by mating *Sirt2^flox/flox^* mice (S-CKO-12757, Cyagen Biosciences, Guangzhou, China) with *Aldh1l1^CreERT2^* mice (I001112, Cyagen Biosciences, Guangzhou, China). Tamoxifen (HY-13757A, MCE) was administered intraperitoneally at 100 mg/kg every 48 h, beginning 3 d before SCI and continuing until tissue collection, to ensure efficient recombination in resident astrocytes and injury-induced glial progenitor-derived astrocytes. Tamoxifen-treated *Sirt2^flox/flox^* littermates served as controls. Mice were maintained under specific-pathogen-free conditions at 23 ± 1 °C and 60% ± 5% humidity on a 12-h light/dark schedule, with free access to chow and sterile water.

### Mouse SCI crush model

Animals were anesthetized with pentobarbital (50 mg/kg, intraperitoneal). After exposing the T10 spinal cord by laminectomy, a moderate crush lesion was produced for 5 s using Dumont No. 5 forceps fitted with a spacer that set the tip width to 0.2 mm and the closed-tip gap to 0.25 mm (Fine Science Tools, 11251-20, Germany) [31]. Successful injury was judged by immediate bilateral hind-limb twitching, a visible compression trace at the lesion, and the absence of hemorrhagic erythema in adjacent cord tissue. Postoperatively, bladders were expressed twice per day, and Baytril (10 mg/kg) was administered subcutaneously once daily for 1 week.

### Sirt2 and lactate dehydrogenase inhibitor treatment

For pharmacological inhibition, AK-7, a brain-penetrant Sirt2 inhibitor [72], was used in vivo. AK-7 (420831-40-9; TargetMol Bioscience Co., Ltd., Shanghai, China) was first dissolved in dimethyl sulfoxide (DMSO; 50 mg/ml; HY-Y0320C, MedChemExpress, Monmouth Junction, NJ, USA) and then diluted immediately before use in a vehicle containing 5% DMSO and 95% corn oil. Eight-week-old WT C57BL/6 mice of both sexes received intraperitoneal AK-7 at 20 mg/kg once daily after SCI until tissue collection. Vehicle-treated mice received the same injection schedule with the corresponding solvent.

To reduce lactate production, *Sirt2* cKO mice received intraperitoneal stiripentol, a lactate dehydrogenase A inhibitor (HY-103392; MedChemExpress, Monmouth Junction, NJ, USA), at 150 mg/kg every other day after SCI until tissue collection.

### Western blot analysis

For western blot analysis, astrocytes were magnetically isolated from spinal cord tissues of WT mice at 0, 3, 7, 14, and 28 dpi. Five biological replicates were analyzed at each time point, with each replicate consisting of astrocytes pooled from 5 mice. Purified astrocytes were lysed in radioimmunoprecipitation assay buffer supplemented with protease and phosphatase inhibitors. Protein concentration was determined by bicinchoninic acid (BCA) assay, and equal protein amounts were denatured, separated by sodium dodecyl sulfate–polyacrylamide gel electrophoresis, and transferred onto polyvinylidene fluoride membranes. After blocking for 2 h in Tris-buffered saline with Tween 20 containing 5% nonfat milk, membranes were incubated overnight at 4 °C with an antibody against Sirt2 (1:1,000; S8447, Sigma). After incubation with the corresponding secondary antibody, bands were visualized by enhanced chemiluminescence. The same membranes were then treated with Primary and Secondary Antibody Stripping Solution (P0025, Beyotime), reblocked, and reprobed with β-actin antibody (1:4,000; 20536-1-AP, Proteintech), followed by the corresponding secondary antibody. For the temporal comparison of Sirt2 expression, samples from all time points were processed under identical experimental conditions, and Sirt2 bands used for quantitative analysis were acquired using the same imaging and exposure settings. Band intensities were quantified using ImageJ, and the Sirt2 signal in each sample was normalized to the corresponding β-actin signal obtained from the same membrane.

### Tissue fixation, sectioning, and immunofluorescence

Mice were transcardially perfused with 40 ml of 0.1 M phosphate-buffered saline (PBS; Biosharp, Hefei, China), followed by 20 ml of 4% paraformaldehyde (PFA; Biosharp). A 1-cm segment centered on the T10 lesion was dissected, fixed in 4% PFA for 5 to 6 h, cryoprotected overnight in 30% sucrose, embedded in optimal cutting temperature compound (SAKURA, 4583), and cut into 16-μm sagittal or horizontal sections. For immunostaining, sections were permeabilized in 0.3% Triton X-100 and blocked with 10% donkey serum at room temperature for 1 h before incubation with primary antibodies at 4 °C overnight [92]. Primary antibodies included rabbit anti-Sirt2 (1:100, S8447, Sigma), rat anti-GFAP (1:400, 13-0300, Invitrogen), chicken anti-GFAP (1:200, 824-8540, Aves Labs), rabbit anti-GFAP (1:100, 16825-1-AP, Proteintech), goat anti-CD31 (1:100, AF3628, R&D Systems), rabbit anti-Pan-lysine lactylation (Pan-Kla, 1:100, PTM-1401, PTM BIO), chicken anti-green fluorescent protein (anti-GFP; 1:500, GFP1010, Aves Labs), goat anti-red fluorescent protein (anti-RFP; 1:200, 200-101-379, Rockland), rat anti-Mac2 (1:200, sc-23938, Santa Cruz), goat anti-Olig2 (1:250, AF2418-SP, Novus), guinea pig anti-NeuN (1:500, SYS-266-004, SYSY), rabbit anti-H4K8la (1:100, PTM-1415RM, PTM BIO), rat anti-C3 (1:250, ab182, Abcam), rabbit anti-S100A10 (1:250, PA5-9550, Invitrogen), rat anti-Ki67 (1:100, 14-5698-80, Invitrogen), rabbit anti-claudin-5 (1:100, 34-1600, Invitrogen), rabbit anti-ZO-1 (1:100, 61-7300, Invitrogen), rat anti-CD68 (1:200, MCA1957, AbD Serotec), goat anti-5-HT (1:100, 20079, Immunostar), and rabbit anti-hepatocyte growth factor (anti-HGF, 1:50, 26881-1-AP, Proteintech). Alexa Fluor 488-, 555-, or 647-conjugated secondary antibodies (1:500, Invitrogen) were applied after washing, and nuclei were counterstained with 4′,6-diamidino-2-phenylindole (D9542, Sigma).

### Microscopy and image-based measurements

Fluorescence images were acquired with a Zeiss Axio Scope A1 microscope and analyzed in TissueFAXS viewer and ImageJ 2.0 (National Institutes of Health, USA). Quantification was performed by investigators blinded to experimental allocation. Unless stated otherwise, each mouse contributed 3 sagittal sections containing the LC, and 5 mice were analyzed in each group.

For microvessel-diameter analysis, GFAP staining was used to define the LC, marginal region, and uninjured region using a modified published strategy [45]. LC corresponded to GFAP-negative tissue within 300 μm of the epicenter, the marginal region corresponded to GFAP-positive peri-lesional tissue located 300 to 500 μm from the epicenter, and uninjured tissue was sampled more than 3 mm from the epicenter. At least 20 CD31-positive vessels with clear signals were selected from each region. To determine the cellular distribution of Sirt2, sections were costained with Sirt2 and lineage markers, including GFP^+^/RFP^−^ for microglia, Mac2 for macrophages, Olig2 for oligodendrocytes, and NeuN for neurons. Three random 40× fields per section were analyzed, and colocalization was reported as the percentage of each lineage among all Sirt2-positive cells.

Sirt2 protein depletion in GFAP-positive cells was evaluated by measuring Sirt2 mean fluorescence intensity (MFI) in 3 random 10× fields per section. The effect of genetic or pharmacological Sirt2 inhibition on lactylation was quantified by measuring Pan-Kla and H4K8la MFI in GFAP-positive astrocytes from the same number of fields; values are expressed as the average fluorescence intensity of Pan-Kla or H4K8la within GFAP-positive astrocytes. Astrocyte proliferation and reactive marker expression were assessed in 3 random 40× fields per section by calculating the proportions of GFAP^+^C3^+^, GFAP^+^S100A10^+^, and GFAP^+^Ki67^+^ cells among all GFAP-positive cells.

For rescue studies, HGF abundance and viral transduction were assessed in sections stained for HGF, GFP, and GFAP. Astrocyte-targeted viral delivery was calculated as GFP^+^GFAP^+^ cells divided by total GFAP-positive cells. HGF expression was quantified as HGF MFI in GFAP-positive cells from 3 random 40× fields per section. NeuN-positive neurons and CD68-positive inflammatory cells were measured in 3 horizontal sections per mouse. Regions were assigned by distance from the LC: Z1, 0 to 250 μm; Z2, 250 to 500 μm; Z3, 1,000 to 1,250 μm; Z4, 2,000 to 2,250 μm; and uninjured control tissue. Z1 to Z4 were sampled discontinuously to avoid overlap and to cover peri-lesional as well as distal tissue. Cell counts were obtained from 10× images, and the mean value was used for each mouse. Descending serotonergic fibers were quantified from 5-HT-stained horizontal sections; the 5-HT-positive area in 5× images was expressed as a fraction of the total tissue area.

### Vessel coverage and BSCB permeability

Endothelial area was assessed with CD31 staining, whereas ZO-1 and claudin-5 were used as tight-junction markers. CD31-positive area was measured in ImageJ with the Measure and Analyze Particles tools and expressed as the percentage of the section occupied by CD31 signal. For tight-junction analysis, images were acquired at the lesion epicenter and penumbra using a 40× objective [93]. Tight-junction coverage was calculated as the number of CD31-positive vessels colabeled with ZO-1 or claudin-5 divided by the total number of CD31-positive vessels.

Barrier permeability was evaluated by EB leakage as previously reported. EB solution (2%, 0.2 ml; Aladdin, China) was injected via the tail vein. After 1 h, mice were euthanized and perfused with saline followed by 4% PFA. A 10-mm spinal cord segment containing the lesion was collected, fixed in 4% PFA, cut into 16-μm sections, and examined for EB fluorescence. EB intensity was quantified in ImageJ.

### Single-cell suspension preparation and scRNA-seq

For scRNA-seq, *Sirt2* cKO mice and *Sirt2^flox/flox^* littermates received T10 crush injury, and spinal cord tissue was collected at 14 dpi. Eight mice were pooled for each group. Animals were euthanized with an overdose of pentobarbital, and a fresh unperfused 1-cm cord segment centered on the lesion was collected rapidly.

NovelBio Co., Ltd. performed the scRNA-seq workflow. Tissues were kept in MACS Tissue Storage Solution (Miltenyi Biotec), rinsed with PBS, minced on ice into approximately 1-mm^3^ fragments, and digested for 30 min at 37 °C with agitation in papain (20 U/ml) and DNase I (30 U/ml). Cell suspensions were filtered through 70- and 40-μm strainers and centrifuged at 400 × *g* for 5 min. After removal of the supernatant, cell pellets were resuspended in red blood cell lysis buffer (Miltenyi Biotec) to remove erythrocytes. Cells were then washed and resuspended for downstream single-cell analysis. Cell viability was assessed by acridine orange/propidium iodide staining using a Countstar Fluorescence Cell Analyzer.

Single-cell transcriptomes were generated with the NovelCyto Single-Cell Analysis System (NovelBio Co., Ltd., China). Cell suspensions were distributed by limited dilution into more than 100,000 microwells, and oligonucleotide-barcoded beads were added until bead saturation. Following cell lysis, captured mRNA was reverse-transcribed on beads and treated with exonuclease I. During complementary DNA (cDNA) synthesis, each cDNA molecule was labeled with a unique molecular identifier and a cell barcode. The resulting cDNA was amplified, fragmented, and converted into sequencing libraries. Library size was checked with an Agilent 4200 bioanalyzer and Qubit High Sensitivity DNA assay, and qualified libraries were sequenced on a DNBSEQ-T7 platform (MGI, Shenzhen, China) using 150-bp paired-end reads.

### scRNA-seq processing

NovelBio Co., Ltd. analyzed the raw scRNA-seq reads on the NovelBrain Cloud Analysis Platform. Adapter sequences and low-quality reads were removed with fastp v0.24.0. STARsolo v2.7.11b was then used to align and quantify reads against the mouse mm10 genome using Ensembl annotation version 100. Cell–cell communication analysis was performed based on known ligand–receptor pairs across annotated cell populations, and predicted interactions were visualized using circle/chord plots.

### Magnetic enrichment of astrocytes

For astrocyte purification, *Sirt2* cKO and *Sirt2^flox/flox^* mice were subjected to T10 crush SCI. At 14 dpi, mice were euthanized with pentobarbital at 1.5 times the surgical dose, and a fresh unperfused 1-cm spinal cord segment centered on the lesion was collected. Cell numbers were determined before magnetic separation. Suspensions were centrifuged at 300 × *g* for 10 min, and pellets were resuspended in sorting buffer (PBS with 0.5% bovine serum albumin) at 80 μl per 1 × 10^7^ cells. Fc receptors were blocked with 10 μl of FcR Blocking Reagent per 1 × 10^7^ cells for 10 min at 4 °C, followed by Anti-ACSA-2 MicroBeads (10 μl per 1 × 10^7^ cells) for 15 min at 4 °C. Cells were washed, resuspended in 500 μl of buffer per 1 × 10^7^ cells, and passed through LS columns. Magnetically retained ACSA-2-positive cells were collected and used for bulk RNA-seq, CUT&Tag, ChIP–qPCR, qPCR, untargeted metabolomics, and Seahorse assays.

### Bulk RNA-seq

Total RNA was isolated with TRIzol reagent (Invitrogen) from purified astrocytes at 14 dpi. Three biological replicates were prepared for each genotype; each replicate contained astrocytes purified from spinal cord tissue pooled from 5 mice. RNA quality was evaluated on an Agilent 2200 instrument, and only samples with an RNA integrity number above 7.0 were used for library construction. Libraries were generated with the VAHTS Universal V6 RNA-seq Library Prep Kit for Illumina (Vazyme, Inc.). Poly(A)^+^ RNA from 1 μg of total RNA was captured with oligo(dT) magnetic beads; fragmented to 200 to 600 bp at 85 °C; reverse-transcribed; processed through end repair, A-tailing, adapter ligation, strand selection, and PCR amplification; and sequenced as 150-bp paired-end reads on a DNBSEQ-T7 platform.

Clean reads were obtained by trimming adapters and filtering low-quality sequences. Reads were mapped to the mouse mm10_Ensembl100 reference with STAR. Gene-level counts were generated with HTSeq, and expression was reported as reads per kilobase of transcript per million mapped reads. DESeq2 identified differentially expressed genes using fold change >2 or <0.5 with *P* < 0.05 and false discovery rate < 0.05. GO enrichment used terms from the National Center for Biotechnology Information, UniProt, and the GO database; KEGG pathway analysis was also performed. Fisher exact testing was used for enrichment analyses, and *P* < 0.05 was considered significant.

### CUT&Tag

CUT&Tag was carried out with the Hyperactive In-Situ Chromatin Immunoprecipitation Library Prep Kit for Illumina (TD901-TD902; Vazyme Biotech, China) according to the kit instructions [31]. Astrocytes purified from *Sirt2* cKO and *Sirt2^flox/flox^* spinal cords at 14 dpi were analyzed in 2 biological replicates per group, with each replicate prepared from 5 pooled mice. Cells were immobilized on concanavalin A-coated magnetic beads, incubated overnight at 4 °C with rabbit anti-H4K8la antibody in antibody buffer, and then incubated with goat anti-rabbit immunoglobulin G (IgG) H&L (AB206-01-AA) for 1 h at room temperature. After protein A-Tn5 transposase treatment, tagmentation was performed for 1 h, and DNA was recovered, amplified, purified, and used for library construction. Libraries were sequenced on an Illumina NovaSeq 6000 platform with 150-bp paired-end reads. DiffBind was used for differential peak calling, and statistically significant differential binding sites were defined as those with *P* < 0.05 and |log_2_(fold change)| > 1, with Benjamini–Hochberg correction applied for multiple testing. Fracher (Wuhan, China) processed raw data, and promoter-region peaks were functionally annotated by GO and KEGG analyses.

### ChIP–qPCR

ChIP–qPCR was performed using astrocytes isolated from *Sirt2* cKO and *Sirt2^flox/flox^* spinal cords at 14 dpi. Three biological replicates were analyzed per group, each generated from 5 pooled mice. Chromatin preparation was performed using the SimpleChIP Enzymatic Chromatin IP Kit (Magnetic Beads; Cell Signaling Technology, 9003) according to the manufacturer’s instructions. Cells were cross-linked with 1% formaldehyde for 10 min at room temperature and quenched with glycine for 5 min. Nuclei were isolated, and chromatin was digested with micrococcal nuclease at 37 °C for 20 min, followed by brief sonication. Diluted chromatin was incubated with H4K8la antibody or normal rabbit IgG overnight at 4 °C. Protein G magnetic beads (30 μl) were added for 2 h, and bound chromatin was washed and eluted. Cross-links were reversed with NaCl and proteinase K at 65 °C for 2 h. DNA was purified using the columns provided with the kit and analyzed by qPCR. The HGF ChIP primers were 5′-TCA CTC TAT GCT GCT TTC CAG G-3′ and 5′-TTG TGT GCC AGT ATT TAG AGG A-3′.

### Quantitative PCR

RNA from tissues or purified cells was extracted with TRIzol reagent (Invitrogen). Each group contained 3 biological replicates, each generated from spinal cord tissue pooled from 5 mice before astrocyte isolation. First-strand cDNA was synthesized using a cDNA synthesis kit (TransGen). qPCR was performed using SYBR Green Pro Taq HS Premix (AG11702) in a 20-μl reaction. The cycling conditions were 95 °C for 30 s, followed by 40 cycles of 95 °C for 5 s and 60 °C for 30 s. The Hgf primers were 5′-GCT CCG CTC TCT TCT TAT GCT-3′ and 5′-ACC AAT TCG CCC CGT TTG AA-3′; the Gapdh primers were 5′-AGG TCG GTG TGA ACG GAT TTG-3′ and 5′-GGG GTC GTT GAT GGC AAC A-3′. Relative expression was normalized to Gapdh.

### Untargeted metabolomics

Untargeted metabolomic profiling was conducted with purified astrocytes. Six biological replicates were included per group, and each replicate came from spinal cord tissue pooled from 5 mice before astrocyte isolation. Frozen samples were thawed on ice, mixed with 500 μl of methanol solution (4:1, v/v) containing internal standard, and vortexed for 3 min. Samples then underwent 3 freeze–thaw/vortex cycles consisting of liquid nitrogen for 5 min, dry ice for 5 min, thawing, and vortexing for 2 min. After centrifugation at 12,000 rpm for 10 min at 4 °C, 300 μl of supernatant was collected, kept at −20 °C for 30 min, and centrifuged again at 12,000 rpm for 3 min at 4 °C, and 200 μl of the supernatant was transferred for liquid chromatography–mass spectrometry (LC–MS).

Samples were analyzed using LC–MS in both positive- and negative-ion modes. Chromatographic separation was performed on a Waters ACQUITY Premier HSS T3 column (1.8 μm, 2.1 mm × 100 mm). Mobile phase A was water containing 0.1% formic acid, and mobile phase B was acetonitrile containing 0.1% formic acid. The gradient was as follows: 5% to 20% B in 1 min, increased to 99% B over the next 2 min and held for 1.5 min, and returned to 5% B within 0.1 min and held for 1.4 min. The column temperature was 40 °C, the flow rate was 0.4 ml/min, and the injection volume was 3 μl. Mass spectra were acquired using electrospray ionization in both positive- and negative-ion modes over an *m*/*z* range of 70 to 1,000 at 60,000 resolution. The spray voltage was 3.8 kV in positive mode and 3.4 kV in negative mode; the sheath gas was 60 arbitrary units, the auxiliary gas was 20 arbitrary units, the ion-transfer tube temperature was 320 °C, the vaporizer temperature was 350 °C, and the collision energies were 30, 40, and 50 V.

Peak areas were corrected using support vector regression, and metabolomic data were normalized to protein content determined by the BCA assay to reduce variation related to differences in sample amount. Unsupervised principal component analysis was performed using the prcomp function in R, with unit variance scaling applied before analysis. Hierarchical cluster analysis and Pearson correlation coefficient (PCC) heatmaps were generated using the R package ComplexHeatmap. For 2-group comparisons, differential metabolites were identified using a variable importance in projection (VIP) value >1 and a nominal *P* value <0.05 based on a Student *t* test. VIP values were derived from orthogonal partial least squares discriminant analysis (OPLS-DA) performed using the R package MetaboAnalystR. Data were log_2_-transformed and mean-centered before OPLS-DA, and model robustness was assessed using 200 permutation tests.

### Seahorse extracellular flux assays

Astrocyte bioenergetics were measured with the Seahorse XFp Real-Time ATP Rate Assay (103591-100), Seahorse XF Cell Mito Stress Test (103015-100), and Seahorse XF Glycolysis Stress Test (103020-100) on an Agilent Seahorse XFe24 analyzer. Five biological replicates were analyzed for each group, with each replicate generated from spinal cord tissue pooled from 5 mice before astrocyte isolation. Purified astrocytes were plated at 200,000 cells per well. Sensor cartridges were hydrated overnight in 1 ml of calibration buffer at 37 °C in a non-CO_2_ incubator, and Seahorse plates were coated with poly-d-lysine and rinsed before use.

For ATP-rate and mitochondrial-stress assays, cells were suspended in XF Dulbecco’s modified Eagle medium containing 10 mM glucose, 1 mM sodium pyruvate, and 2 mM l-glutamine. For glycolysis-stress assays, cells were placed in Seahorse XF Base Medium containing 2 mM l-glutamine. Plates were centrifuged at 2,000 × *g* for 20 min at 4 °C and equilibrated for 20 min at 37 °C without CO_2_. ATP production was assessed after sequential addition of oligomycin (15 μM, 56 μl) and rotenone/antimycin A (5 μM, 62 μl). Oxygen consumption rate was measured with oligomycin (15 μM, 56 μl), carbonyl cyanide 4-(trifluoromethoxy) phenylhydrazone (10 μM, 62 μl), and rotenone/antimycin A (5 μM, 69 μl). Extracellular acidification rate was measured with glucose (100 mM, 56 μl), oligomycin (10 μM, 62 μl), and 2-deoxy-d-glucose (500 mM, 69 μl). Glutamine oxidation was examined in substrate-free base medium containing 4 mM glutamine as described previously. After assays, plates were centrifuged at 2,000 × *g* for 10 min at 4 °C, the protein in each well was measured with the Pierce BCA Protein Assay (Thermo Fisher Scientific, 23225), and flux data were normalized to total protein and analyzed in Seahorse Wave Desktop Software v2.6.1.

### AAV-mediated Hgf knockdown

To reduce astrocytic HGF expression, AAV-GFaABC1D-EGFP-5′miR-30a-shRNA1(*Hgf*)-3′miR30a-WPREs (AAV-sh*Hgf*; Shumi Brain Science and Technology, Wuhan, China) was used. AAV-GFaABC1D-EGFP-5′miR-30a-shRNA(scramble)-3′miR30a-WPREs served as the control vector. AAV was injected into the T10 spinal cord parenchyma at the intended injury site 35 d before SCI. A total of 5 × 10^9^ vg viral particles in 1 μl were injected at a depth of 1 mm and a rate of 200 nl/min using a 10-μl Hamilton syringe connected to a neMESYS OEM microsyringe pump controller (Cetoni, Germany).

### Locomotor assessment

BMS scores were recorded at 0, 3, 7, 14, 21, and 28 dpi. Three trained observers who were blinded to treatment group evaluated ankle movement, paw placement, coordination, trunk stability, and tail position. Scores ranged from 0 to 9, with higher values indicating better hind-limb locomotor function.

### Statistical analysis

Animals were randomly assigned to experimental groups. Investigators were blinded to genotype or treatment during behavioral testing, image acquisition/quantification, and statistical analysis whenever possible. No animals were excluded unless predefined criteria were met, including failed injury induction or postoperative death. Sample sizes were chosen based on previous studies and pilot experiments; no formal prospective sample size calculation was performed.

Data are shown as mean ± standard error of the mean unless otherwise specified. GraphPad Prism 8.0 was used for statistical testing and graph preparation. Pearson correlation analysis was used for relationships between 2 variables. For multiple-group comparisons, 1-way or 2-way analysis of variance followed by Tukey’s post hoc test was applied as appropriate. Two-group comparisons were performed with a 2-tailed unpaired Student *t* test. A value of *P* < 0.05 was considered statistically significant. Significance is indicated as *P < 0.05, **P < 0.01, ***P < 0.001, and ****P < 0.0001.

## Data Availability

The data supporting this study are included in the article and Supplementary Materials. Sequencing data have been deposited in the National Genomics Data Center (NGDC) under accession numbers CRS041432 for bulk RNA-seq, CRS041408 for CUT&Tag, and CRS041493 for scRNA-seq. Untargeted metabolomics data are available under accession number OMIX016238.
