# Supplementary material for: Astrocytic Sirt2-Mediated Delactylation Limits Blood–Spinal Cord Barrier Reconstruction after Spinal Cord Injury
Source: Research (Wash D C). 2026 Sep 30;9:1447. doi: 10.34133/research.1447 (PMC13624570; doi:10.34133/research.1447)
Supplement: Supplementary file 1 [file research.1447.f1.docx]

**SUPPLEMENTARY MATERIALS**

**Supplementary Fig. 1.** **Sirt2 is enriched in astrocytes among candidate delactylases after SCI.** (A) Dot plot of canonical markers used to annotate major cell populations in a published SCI scRNA-seq dataset. (B) UMAP of reclustered astrocytes from the same dataset. (C) Feature plots of candidate delactylases, including Sirt1, Sirt2, Sirt3, Hdac1, Hdac2, and Hdac3, in astrocytes. (D) Temporal feature plot of astrocytic Sirt2 expression before injury and at the indicated dpi.

**Supplementary Fig. 2. Generation and baseline characterization of astrocyte-specific Sirt2 conditional knockout mice.** (A) Body-weight curves of control and *Sirt2* cKO mice. (B) Footprint images; forepaws are shown in green and hindpaws in red. (C, D) Quantification of stride width and stride length from B. (E) Heatmap of significantly altered metabolites in purified astrocytes from control and *Sirt2* cKO mice at 14 dpi. Data are mean ± s.e.m.; n = 8 for A, C, and D; n = 6 for E. Two-way ANOVA with Tukey’s test was used for A; two-tailed unpaired Student’s t test was used for C, D, and E. ns, not significant.

**Supplementary Fig. 3. Marker gene expression in major spinal cord cell populations after SCI.** (A) Dot plot showing representative canonical marker genes for the major cell populations identified by scRNA-seq at 14 dpi.

**Supplementary Fig. 4. Annotation of the major populations identified by scRNA-seq.** (A–K) Feature plots of representative markers used for cell-type annotation: *P2ry12* for microglia, *Spp1* for macrophages, *Olig1* for oligodendrocyte-lineage cells, *H2-Eb1* for monocytes, *Ms4a4b* for lymphocytes, *Ly6c1* for endothelial cells, *Sox9* for astrocytes, *Col3a1* for fibroblasts, *Rgs5* for pericytes, and *S100a8* for neutrophils.

**Supplementary Fig. 5. Astrocytic Sirt2 deletion alters lesion cell composition and transcriptional programs after SCI.** (A, B) UMAP plots of major cell populations in control and *Sirt2* cKO spinal cords at 14 dpi. (C) Relative abundance of major cell types in the two groups, including lymphocytes, endothelial cells, and astrocytes. (D) UMAP of reclustered astrocytes from control and *Sirt2* cKO mice. (E) Volcano plot of differentially expressed genes in *Sirt2* cKO astrocytes. (F) Dot plot of selected A1- and A2-associated genes. (G, H) GO and KEGG enrichment analyses of genes upregulated in *Sirt2* cKO astrocytes by scRNA-seq. (I) KEGG analysis of genes upregulated in MACS-purified *Sirt2* cKO astrocytes by bulk RNA-seq.

**Supplementary Fig. 6. LDH inhibition attenuates Sirt2 deletion-associated H4K8la accumulation and reactive astrocyte marker changes after SCI.** (A) H4K8la (purple), GFAP (green), and DAPI (blue) staining and quantification of H4K8la fluorescence intensity in GFAP-positive astrocytes from *Sirt2^flox/flox^* + DMSO, *Sirt2* cKO + DMSO, and *Sirt2* cKO + stiripentol (STP) mice. Scale bar, 50 μm. (B) C3 (purple), GFAP (green), and DAPI (blue) staining and quantification of GFAP-positive C3-positive astrocytes. Scale bars, 100 μm for overview images and 20 μm for insets. (C) S100A10 (purple), GFAP (green), and DAPI (blue) staining and quantification of GFAP-positive S100A10-positive astrocytes. Scale bars, 100 μm for overview images and 20 μm for insets. Data are mean ± s.e.m.; *n* = 5 mice per group. One-way ANOVA with Tukey’s test was used. ****P < 0.0001.

**Supplementary Fig. 7. Endothelial subpopulation analysis after astrocytic Sirt2 deletion.** (A) Cell–cell communication circle plot based on ligand–receptor analysis of the single-cell RNA-sequencing dataset, showing predicted interactions between astrocytes and endothelial cells. The colored sectors denote the corresponding cell populations, while pink and blue indicate receptors and ligands, respectively. The color scale labeled “Mean” represents the mean expression level associated with the corresponding ligand–receptor interaction, and the connecting lines indicate predicted intercellular communication events. (B, C) Endothelial reclustering and marker-gene expression. (D, E) Endothelial subpopulation distribution in control and *Sirt2* cKO mice.

**Supplementary Fig. 8. Bioinformatic analysis of differential promoter-associated peaks.** (A) GO enrichment of genes linked to differential promoter peaks. (B) KEGG enrichment of genes associated with differential promoter peaks.

**Supplementary Fig. 9.** **Validation of AAV-mediated astrocytic Hgf knockdown in vivo.** (A) GFP (green), GFAP (purple), and DAPI (blue) staining in spinal cord sections from the indicated groups, with quantification of GFP^+^GFAP^+^ cells among total GFAP^+^ cells. Scale bar, 50 μm. (B) HGF (green), GFAP (purple), and DAPI (blue) staining, with quantification of HGF fluorescence intensity in GFAP^+^ cells. Scale bar, 50 μm. Data are mean ± s.e.m.; n = 5 for A and B. One-way ANOVA with Tukey’s test was used. ns, not significant; ****P < 0.0001.

**Supplementary Fig. 10. Astrocytic Sirt2 deletion remodels lymphocyte composition after SCI.** (A) UMAP plot of reclustered lymphocytes showing CD4^+^ T cells, CD8^+^ T cells, and B cells. (B) Dot plot of canonical lymphocyte markers, including *Cd4*, *Cd3d*, *Cd8a*, *Cd8b1*, *Ly6d*, *Cd79a*, and *Cd79b*. (C, D) Pie charts or bar plots showing the proportions of CD4^+^ T cells, CD8^+^ T cells, and B cells in control and *Sirt2* cKO spinal cords. (E) Astrocyte–CD8^+^ T cell interaction analysis showing representative ligand–receptor pairs, including Cd55-Adgre5, Fas-Fasl, Cntn1-Notch2, Sema4d-Ptprc, and collagen-integrin interactions. (F) UMAP plot of shared and genotype-specific lymphocyte populations in control and *Sirt2* cKO mice. (G) Violin plots of cytotoxic and regulatory lymphocyte genes, including *Gzma*, *Gzmb*, *Nkg7*, *Il2rb*, *Gata3*, and *Ikzf2*. (H, I) GO and KEGG enrichment analyses of genes downregulated in lymphocytes from *Sirt2* cKO mice.

**Supplementary Fig. 11. Working model summarizing how astrocytic Sirt2-dependent delactylation regulates BSCB reconstruction after SCI.**
